# Noninvasive Stratification of Colon Cancer by Multiplex PET Imaging

**DOI:** 10.1158/1078-0432.CCR-23-1063

**Published:** 2024-03-17

**Authors:** Gaurav Malviya, Tamsin R.M. Lannagan, Emma Johnson, Agata Mackintosh, Robert Bielik, Adam Peters, Dmitry Soloviev, Gavin Brown, Rene Jackstadt, Colin Nixon, Kathryn Gilroy, Andrew Campbell, Owen J. Sansom, David Y. Lewis

**Affiliations:** 1 Cancer Research UK Scotland Institute, Garscube Estate, Glasgow, United Kingdom.; 2 School of Cancer Sciences, University of Glasgow; Glasgow, United Kingdom.; 3 Heidelberg Institute for Stem Cell Technology and Experimental Medicine (HI-STEM gGmbH), Heidelberg, Germany.; 4 Cancer Progression and Metastasis Group, German Cancer Research Center (DKFZ), and DKFZ-ZMBH Alliance, Heidelberg, Germany. German Cancer Consortium (DKTK), Germany.

## Abstract

**Purpose:**

The current approach for molecular subtyping of colon cancer relies on gene expression profiling, which is invasive and has limited ability to reveal dynamics and spatial heterogeneity. Molecular imaging techniques, such as PET, present a noninvasive alternative for visualizing biological information from tumors. However, the factors influencing PET imaging phenotype, the suitable PET radiotracers for differentiating tumor subtypes, and the relationship between PET phenotypes and tumor genotype or gene expression–based subtyping remain unknown.

**Experimental Design:**

In this study, we conducted 126 PET scans using four different metabolic PET tracers, [^18^F]fluorodeoxy-D-glucose ([^18^F]FDG), O-(2-[^18^F]fluoroethyl)-l-tyrosine ([^18^F]FET), 3′-deoxy-3′-[^18^F]fluorothymidine ([^18^F]FLT), and [^11^C]acetate ([^11^C]ACE), using a spectrum of five preclinical colon cancer models with varying genetics (BMT, AKPN, AK, AKPT, KPN), at three sites (subcutaneous, orthograft, autochthonous) and at two tumor stages (primary vs. metastatic).

**Results:**

The results demonstrate that imaging signatures are influenced by genotype, tumor environment, and stage. PET imaging signatures exhibited significant heterogeneity, with each cancer model displaying distinct radiotracer profiles. Oncogenic *Kras* and *Apc* loss showed the most distinctive imaging features, with [^18^F]FLT and [^18^F]FET being particularly effective, respectively. The tissue environment notably impacted [^18^F]FDG uptake, and in a metastatic model, [^18^F]FET demonstrated higher uptake.

**Conclusions:**

By examining factors contributing to PET-imaging phenotype, this study establishes the feasibility of noninvasive molecular stratification using multiplex radiotracer PET. It lays the foundation for further exploration of PET-based subtyping in human cancer, thereby facilitating noninvasive molecular diagnosis.

**Translational Relevance**

Patient stratification is a crucial step toward implementing personalized treatment for patients with colon cancer. Gene expression profiling has significantly improved the stratification of patients; however, the major limitation of genomic technologies is their dependence on tissue samples. In this study, we introduce a novel imaging approach using multiplexed PET radiotracer imaging in a diverse range of colon cancer mouse models. We systematically investigate the impact of tumor genetics, environment, and stage on tumor phenotypes, aiming to compare and contrast the impact of tumor intrinsic and extrinsic factors on PET imaging signatures. With the advent of low-dose total-body PET imaging technology, the application of multi-radiotracer PET tracers in clinical populations is more achievable. The facile categorization of tumor molecular features into distinct biological groups will aid the targeted administration of new treatments for patients with colon cancer.

## Introduction

Colorectal cancer, the second leading cause of cancer-related deaths, poses a substantial challenge due to its inherent tumor heterogeneity, resulting in varied responses and outcomes among patients (1–3). To improve therapeutic approaches, it is crucial to develop a comprehensive understanding of the biological and diagnostic distinctions between subtypes of colon cancer. In response to this imperative, the community collaborated to create the consensus molecular subtypes (CMS), which classify colorectal cancer into four distinct subtypes based on transcriptional profiling. Each subtype possess prognostic value and exhibits unique characteristics. For example, CMS1 is characterized by microsatellite instability (MSI), CMS2 demonstrates canonical activation of WNT pathways, CMS3 is associated with metabolic dysregulation, and CMS4 is marked by stromal infiltration (4).

The identification of subtypes in colorectal cancer aims to facilitate effective interventions specific to each subtype, similar to the use of BRAF inhibitors for BRAF^V600E^ mutant tumors and EGFR inhibitors for wild-type RAS tumors (5). The development of stratified treatment using CMS profiling is ongoing, and early signs indicate that in the neoadjuvant setting, CMS2 and CM3 groups respond better to 5FU chemotherapy compared with CMS4 and CMS1 groups, which, due to MSI, are more responsive to checkpoint inhibition (6). However, current methods for profiling and stratification rely heavily on invasive procedures such as tumor biopsies or surgical resection. Although biopsies are essential for acquiring tumor samples, they are associated with limitations including sampling bias and potential complications such as bleeding, inflammation, and tumor seeding. These factors can adversely impact prognosis (7–10). Furthermore, obtaining biopsies or surgically resected tissues may not be feasible, particularly at metastatic sites (11). Liquid biopsies for circulating tumor DNA are less invasive but only provide information on tumor genotype and may not inform on tumor phenotype.

To overcome these limitations, noninvasive molecular diagnosis with PET is a promising alternative. Although the radiotracer [^18^F]fluorodeoxy-D-glucose ([^18^F]FDG) is commonly utilized in the staging of colorectal tumors due to high glucose uptake, other PET tracers targeting specific molecular tumor features, even without universal uptake, may effectively distinguish between clinically actionable subtypes (12). Recent advancements in PET radiotracers have demonstrated their superior ability to predict treatment response compared with conventional IHC methods (11, 13, 14). Although highly multiplexed PET imaging studies may not be practical in clinical settings, the availability of multiple PET tracers beyond [^18^F]FDG opens up new possibilities for clinical use. Technologies like total body PET, which require lower radioactive doses, offer opportunities for limited tracer multiplexing (15). However further investigation is needed to determine the complementarity, implementation and selection of these new agents and stratification strategies.

Incorporating PET imaging into the molecular diagnosis of colorectal cancer necessitates a comprehensive understanding of the intrinsic and extrinsic factors that influence imaging phenotype. Previous studies have identified certain tumor factors that associated with specific PET tracer uptake patterns. For instance, mutations in *KRAS* or loss of the tumor suppressor *p21* have been linked to increased [^18^F]FDG and decreased 3′-deoxy-3′-[^18^F]fluorothymidine ([^18^F]FLT) uptake, respectively (16, 17). The tumor microenvironment and its various components also contribute to changes in PET phenotype. For example, hypoxia and reduced perfusion gradients lead to metabolic heterogeneity and increased [^18^F]FDG and [^18^F]fluoromisonidazole ([^18^F]FMISO) uptake (18, 19). In addition, as tumors grow, spread, and metastasize, they undergo biological changes, resulting in increased [^18^F]FDG uptake, which can be used for tumor staging (20, 21). Establishing the connection between tumor factors and imaging phenotypes can be a powerful approach. For instance, the association between [^18^F]FDG PET and *KRAS* mutations has been suggested as a means to select patients for EGFR inhibitor treatment. However, the accuracy of FDG in this regard is insufficient for widespread use, and it is possible that other PET tracers or additional genetic or transcriptional features might enhance our ability to effectively distinguish between different aspects or characteristics of colorectal cancer (22). Fundamentally, PET phenotypes are influenced by complex interactions, and our understanding of the relative contributions of genetics, transcription, microenvironment, and stage to the multitude of PET imaging phenotypes remains incomplete.

To gain insights into the biological factors influencing PET imaging signatures, our study generated 126 PET imaging profiles using four PET radiotracers in state-of-the-art colon cancer organoid models generated using five genetically engineered mouse models (GEMM) that reflect the genetic drivers found in human patients with colon cancer. We identified the most effective radiotracers for separation and determined the most distinctive transcriptional and genetic features. By employing various cancer models and implantation sites, we independently assessed the impact of tumor genetics, tumor microenvironment, and metastatic evolution on PET imaging signatures. The use of PET imaging for cancer characterization offers complementary advantages to molecular profiling, enabling the noninvasive identification of spatial heterogeneity, tumor evolution, and posttherapy subtype switching. This study systematically examined the effect of tumor and environmental factors on PET imaging phenotypes, contributing to a better understanding of the major determinants of PET imaging signatures in colon cancer and paving the way for the use of PET as a tool for patient stratification.

## Materials and Methods

### *In vivo* mouse models

All experiments were performed according to the UK Home Office regulations (project license No. 70/8646) with approval from the local ethical review committee of the University of Glasgow (AWERB). There was no group randomization nor researcher blinding to genotype.

### GEMM

Mouse models were derived from nine alleles as described previously: *Villin^CreER^*, *Apc^fl/+^* (23), *Kras^G12D/+^* (24), *Rosa26^N1icd/+^* (25), *Mlh1^fl/fl^* (26), *Braf^V600E/+^* (27), *Tgfbr1/Alk5 ^fl/fl^* (28), *Tgfbr2^fl/fl^* (29), and *Trp53^fl/fl^* (30). We utilized six GEMM: *Apc^fl/+^* (A), *Apc^fl/+^ Kras^G12D/+^* (AK), *Braf^V600E/+^ Mlh1^fl/fl^ Tgfbr2^fl/fl^* (BMT), *Apc^fl/+^ Kras^G12D/+^ Trp53^fl/fl^ Tgfbr1/Alk5 ^fl/fl^* (AKPT), *Apc^fl/+^ Kras^G12D/+^ Trp53^fl/fl^ Rosa26^N1icd/+^* (AKPN), and *Kras^G12D/+^ Trp53^fl/fl^ Rosa26^N1icd/+^* (KPN; refs. 26, 31, 32). We induced tumors in male and female (6–12 weeks old) mice by recombination of *Villin^CreER^* via 80 mg/kg intraperitoneal injection of tamoxifen (Sigma-Aldrich, T5648). Mice were aged until they showed clinical signs such as anemia, hunching, or weight loss. The GEMM were used for RNA transcriptional analysis, except for the KPN, which was imaged with [^18^F]FDG PET/MRI. Details of all mice used in these studies are presented in Supplementary Table S1.

### Colon cancer organoids

We utilized six different mouse-derived colon cancer organoid lines from five GEMM, as described previously (31). One organoid line per genotype (AK, BMT, AKPT, and AKPN and a KPN primary and metastasis pair) were used in imaging studies (Supplementary Table S1). Organoids were grown in growth factor-reduced Matrigel (Corning, Catalog No. 356231) in a 3D system (15). Advanced DMEM/F12 (Invitrogen, Catalog No. 12634–028) was supplemented with 2 mmol/L glutamine, 10 mmol/L HEPES, and 100 U/mL penicillin/streptomycin (Thermo Fisher Scientific, 15140122), B27 (Invitrogen, Catalog No. 12587–010), and N2-supplement (Thermo Fisher Scientific, 17502001). For culturing organoids, 100 ng/mL Noggin (Peprotech, Catalog No. 250–38), 500 ng/mL R-spondin (R&D Systems, Catalog No. 3474-RS), and 50 ng/mL EGF (Peprotech, Catalog No. AF-100–15) were added. Organoids were cultured in six-well plates (BD Falcon) at 37°C in an atmosphere containing 5% CO_2_ and 95% air.

### Implantable tumor models

For subcutaneous tumors, CD-1 nude male mice (approximately 6 weeks old) were purchased from Charles River Laboratories Inc. CD-1 nude mice, which lack functional T cells, were used to improve implantation rates of the organoid lines on various genetic backgrounds. All mice were housed in a specific pathogen-free facility in individually ventilated cages (IVC) for 1 week for acclimatization. Tumor allografts with all six organoid lines; AK (*n* = 5), BMT (*n* = 6), AKPT (*n* = 5), AKPN (*n* = 5), KPN (*n* = 5) colon tumor organoids, and a matched KPN liver metastasis (*n* = 5) organoid were induced by subcutaneous injection into the right shoulder blade of mice (50 organoids/mouse) in a 100-μL volume (Supplementary Fig. S2). After allograft transplantation, the mice were monitored for tumor growth, weight loss, paling feet, and any other clinical symptoms three times per week. Tumor allografts were grown for approximately 2 weeks before PET/MR imaging. The same subcutaneous KPN implanted mice (*n* = 5) were used as a reference in comparisons between models, implantation sites, and between primary and metastasis. For orthograft tumors, male C57BL/6 mice approximately 6 weeks old (Charles River Laboratories, Inc.) were acclimatized for 1 week. C57BL/6 mice were used for the orthotopic model as the AKPT and KPN organoids were derived from mice on a pure C57BL/6 background. Mice were anesthetized with isoflurane and tumor orthografts induced by intracolonic injection of colon tumor organoids, KPN (*n* = 4) and AKPT (*n* = 4) in 70-μL volume using a Hamilton syringe (Hamilton Inc.). The injection needle was positioned on the colon mucosa, with the bevel facing the mucosa. The organoids were injected into the mucosa to form a bubble. Tumor growth was monitored by colonoscopy every 2 weeks posttransplantation. Colonoscopy was performed using a Karl Storz Tele Pack Vet X LED endoscopic video unit (Karl Storz SE & Co. KG) to monitor tumor growth in the colon. Tumor orthografts grew for about 3 to 4 weeks before PET/MR imaging. Mice were not randomized for tumor implantations, nor were the researchers blinded to the models. Details of all mice used in these studies are presented in Supplementary Table S1.

### Transcriptional profiling and analysis

Primary colon tumors were sampled from the intestines of genetically engineered mice: A (*n* = 5), AK (*n* = 1), AKPN (*n* = 3), BMT (*n* = 5), AKPT (*n* = 4), and KPN (*n* = 18). Total RNA was extracted using the Qiagen RNeasy Kit (74104), according to the manufacturer's instructions. Libraries for cluster generation and DNA sequencing were prepared following an adapted method from the Illumina TruSeq RNA LT Kit. The libraries were run on the Illumina NextSeq 500 using the High Output (75 cycles) Kit (2 × 36 cycles, paired-end reads, single index) with normalization and quality control checks, as described previously (26, 31, 32). CMS calls were performed using the CMScaller package in R (version 2.0.1) using the Nearest Template Prediction algorithm (33). Gene set enrichment scores were generated using the GSVA package in R (version 1.44.3) with the HALLMARK gene list (34).

### PET radiotracer synthesis

All reagents and materials were purchased from Merck Life Science UK Ltd., unless stated otherwise. All radionuclides for radiotracer synthesis were produced with a 16.4-MeV proton beam on the GE Healthcare PETtrace cyclotron at the Radiopharmaceutical Unit (RPU) of the West of Scotland PET Centre, Gartnavel Hospital, Glasgow. [^18^F]FDG was supplied directly by the RPU and had an average volumic activity (*A*_v_) of 0.897 ± 0.014 GBq/mL at the reference time.

Fully automated syntheses of fluorine-18 labeled tracers were carried out on a TRACERLAB FX_FN_ (GE Healthcare) according to previously published procedures. To synthesize O-(2-[^18^F]fluoroethyl)-l-tyrosine ([^18^F]FET) and [^18^F]FLT, no carrier-added [^18^F]fluoride was obtained through the ^18^O(p, n)^18^F nuclear reaction by irradiation of 95–97 atom % ^18^O enriched water (purchased from Sercon UK Ltd.) in a niobium target chamber (2.7 mL target volume). Typically, 80 μA, 15 minutes of target irradiation gave 50 GBq of [^18^F]fluoride at the end of bombardment (EOB) for use at the start of synthesis (SOS). [^18^F]FET was obtained from the O-(2-tosyloxyethyl)-N-trityl-L-tyrosine precursor (ABX GmbH), as described by Hamacher and colleagues (35) with some modifications, namely 1 mL of Kryptofix solution [K2.2.2 (120 mg, 0.319 mmol)/K_2_CO_3_ (22 mg, 0.159 mmol)/CH_3_CN (1 mL)/H_2_O (14 mL)] as the phase transfer catalyst was used instead of tetrabutylammonium hydrogencarbonate, deprotection was carried out with 1 mL of 1 M HCl mixed with 0.5-mL ethanol at 130°C, followed by neutralization with 1-M sodium hydroxide and filtration on an alumina N cartridge (Waters) prior to HPLC purification. Average radiochemical yield (RCY) was 53 ± 24%, *A*_v_ 2.2 ± 1.2 GBq/mL at reference time, radiochemical purity (RCP) >98%. [^18^F]FLT was synthesized from 10 mg of 3-N-Boc-1-[5-O-(4,4′-dimethoxytrityl)-3-O-nosyl-2-deoxy-b-D-lyxofuranosyl]thymine precursor (ABX GmbH) in 1-mL acetonitrile, as described by Suehiro and colleagues (36). RCY 10 ± 7%, *A*_v_ 0.77 ± 0.3 GBq/mL at reference time, RCP >99%.

No-carrier-added [^11^C]carbon dioxide was produced via the ^14^N(p,α)^11^C nuclear reaction by irradiation of an aluminum target filled with 99.9% nitrogen/0.1% oxygen gas mixture purchased from BOC. Typically, at 44 μA, a 50-minute target irradiation of 105 GBq of [^11^C]CO_2_ was obtained at the EOB. [^11^C]Acetate was produced according to Soloviev and Tamburella (37) on a customized SYN THRA module (Synthra GmbH) RCY 55 ± 25%, *A*_v_ 4.02 ± 1.6 GBq/mL at reference time, RCP >95%.

### PET/MRI imaging

PET and MRI scans were performed sequentially with different radiotracers in colon tumor-bearing mice using a nanoScan PET/MRI scanner (Mediso Medical Imaging Systems). Mice were maintained under inhaled isoflurane anesthesia (induction 5% v/v; maintenance 1.5% to 2.0% v/v) in medical air during injection and PET/MRI imaging procedure periods, as described previously [52]. Each subcutaneous allograft-bearing mouse: AK (*n* = 5), BMT (*n* = 6), AKPT (*n* = 5), AKPN (*n* = 5), KPN (*n* = 5), and KPN liver metastasis (*n* = 5) underwent four PET/MR imaging sessions using different PET imaging biomarkers [i.e., [^18^F]FDG, [^18^F]FET, [^18^F]FLT and [^11^C]ACE]. Ten scans were unavailable due to mouse death or tumor ulceration at an earlier time point. Each orthograft tumor-bearing mouse, KPN (*n* = 4) and AKPT (*n* = 4), underwent two PET/MR imaging sessions using different PET imaging biomarkers [i.e., [^18^F]FDG, [^18^F]FET]. The KPN autochthonous model (*n* = 4) underwent a single FDG [^18^F]PET/MR. Details of all mice used in these studies are presented in Supplementary Table S1. A minimum recovery period of at least 24 hours was provided between two consecutive imaging sessions. On average, there was a 3-day gap between the scans. To obtain a uniform injection volume of 200 μL for each radiotracer in each mouse, PET tracers were diluted with 0.9% isotonic saline solution according to the volumic activity of every radiotracer at the end of each radiosynthesis. Each mouse received 15.95 ± 2.1 MBq of [^18^F]FDG, 15.97 ± 1.4 MBq of [^18^F]FET, 13.8 ± 3.59 MBq of [^18^F]FLT, or 252.94 ± 69.2 MBq of [^11^C]ACE, via an intravenous bolus injection in the tail vein. A 20-minute static PET acquisition was performed from 80 to 100 minutes for [^18^F]FDG, [^18^F]FLT, and [^11^C]ACE, and from 50 to 70 minutes for [^18^F]FET. Whole-body T1-weighted Gradient Echo 3D Axial MRI Sequences (slice thickness, 0.6 mm; repetition time, 17.2 milliseconds; echo time, 3.8 milliseconds; flip angle, 30°) were used to acquire MRI scans.

PET scans were reconstructed with Tera-Tomo 3D software (Mediso Medical Imaging Systems) using static, whole-body mode with four iterations, six subsets, body-air-threshold (BAT) 25%, coincidence mode 1 to 3, and an energy window 400 to 600 keV, producing a 0.4-mm isotropic matrix. PET data were corrected for random coincidences, attenuation, scatter, radioactivity decay, and dead time. Scatter and attenuation correction used the whole-body T1‐weighted Gradient Echo 3D images. The reconstructed PET scans were co-registered with MRI scans for anatomical reference.

For quantitative assessment of scans, the volume-of-interest (VOI) was manually drawn around the tumors on MRI scans by visual inspection using PMOD software version 3.504 (PMOD Technologies Ltd.), and the same VOI was copied onto the respective PET scans. Separate areas of interest were drawn for each scan to adjust for the position of the mice on the scanner and the tumor size. Standardized uptake values (SUV) were determined by dividing the radiotracer concentration in the VOI by the injected dose divided by the animal weight. SUV_mean_ was calculated using the average of all voxels within the tumor VOI, whereas SUV_peak_ values were calculated using the mean of the five hottest VOI pixel values.

### IHC and ISH

IHC and ISH staining were performed on 4-μm formalin-fixed paraffin-embedded sections (FFPE) that had previously been heated at 60°C for 2 hours. IHC staining for αGlut-1 (GT12-A, Alpha Diagnostics) was performed using a Leica Bond Rx autostainer. The FFPE sections underwent on-board dewaxing (AR9222, Leica) and antigen retrieval using ER2 solution (AR9640, Leica) for 30 minutes at 100°C. The sections were rinsed with Leica wash buffer (AR9590, Leica) before peroxidase blocking was performed using an Intense R Kit (DS9263, Leica) for 5 minutes. The sections were rinsed with wash buffer before application of the primary antibody at a 1/250 dilution (αGlut-1, 1/250) for 30 minutes. The sections were rinsed with wash buffer, and rabbit EnVision secondary antibody was applied for 30 minutes. The sections were rinsed with wash buffer, visualized using DAB, and counterstained with hematoxylin using the Intense R Kit. ISH detection of *Slc7a5* (472578), Mm-*Ppib* (313918), and dapβ (312038; Bio-Techne) mRNA was performed using the RNAScope 2.5 LSx (Brown) Detection Kit (322700; Bio-Techne) on a Leica Bond Rx autostainer, according to the manufacturer's instructions. To complete ISH staining, the sections were rinsed in tap water, dehydrated through graded ethanol, and placed in xylene. The stained sections were coverslipped in xylene using DPX mountant (SEA-1300–00A; CellPath). IHC and ISH expression was quantified using HALO software (Indica Labs). For IHC, the *H*-score [*H*-score = Σ*P_i_* (*i* + 1), where *P_i_* represents the percentage of stained cells for each intensity *i*, ranging from 0 to 3+] was calculated, and the RNA copies per μm^2^ are presented for ISH.

### Statistical analyses

Heatmaps were plotted using Morpheus (Broad Institute) and grouped using hierarchical clustering, one-minus Pearson correlation, and average linkage on rows and columns. PET imaging matrix was analyzed using the factoextra package in R to create PCA biplots with confidence thresholds. Statistical analysis was performed using GraphPad Prism version 9.5.0 (GraphPad Software). Student *t* tests, two-way ANOVA, and receptor operator characteristic curve tests were used to compare the means of different groups. A significance threshold of *P* < 0.05 was utilized for hypothesis testing.

### Data availability

PET/MR imaging data, histology data, and other data generated in this study are available upon request to the corresponding author. RNA-sequencing data are available using accession numbers GSE245277, GSE167008, and GSE218776.

## Results

### A spectrum of mouse models of colon cancer covers the genotypic and phenotypic diversity of the human disease

**Figure 1.**
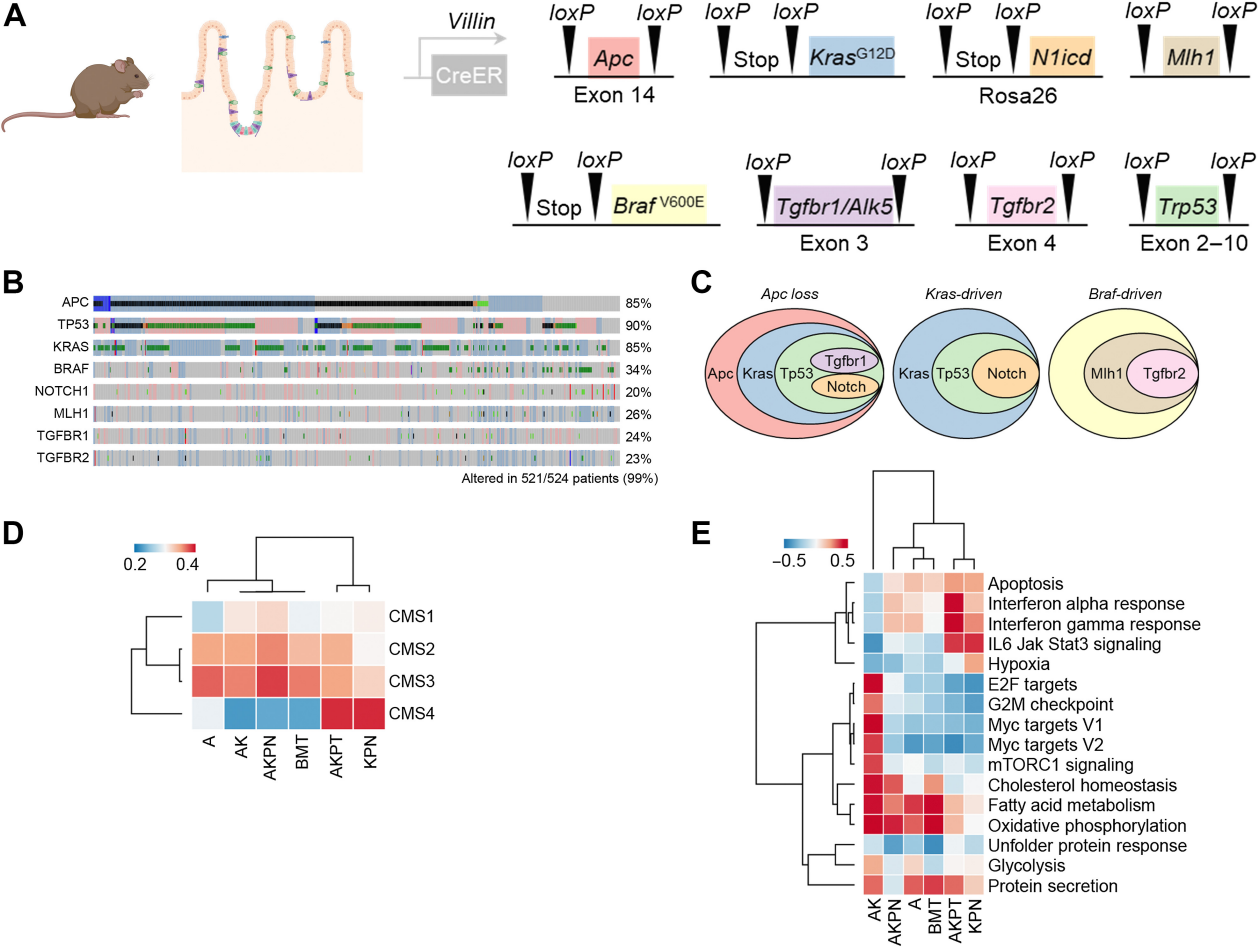
Colon cancer models have genotypic and phenotypic diversity. **A,** The alleles in the GEMM used in this study. Cre, Cre recombinase; ER, estrogen receptor; loxP, Cre-loxP recombination site. **B,** Oncoprint showing the prevalence of genetic alterations of the alleles in **A**, determined using cBioportal from the Colorectal Adenocarcinoma, TCGA, PanCancer Atlas (*n* = 524). **C,** Venn diagrams of the genetic crossing strategies for the GEMM. **D,** Heatmap illustrating correlation of intestinal cancer GEMM gene expression with patient-derived CMS profiles. **E,** Gene set enrichment analysis for the GEMM with the Molecular Signatures Database (MSigDB) hallmark gene set. Data were ordered using hierarchal clustering. Extended heatmap is shown in Supplementary Fig. S1. (**A**, Created with BioRender.com.)

Colon cancer is a molecularly diverse disease with different genetic and phenotypic features that contribute to inter- and intratumor heterogeneity (3, 4, 38). To model the breadth of human colon cancer, we used eight conditionally induced tumor suppressors or oncogenic alleles activated in the intestinal epithelia by Villin-CreER (Fig. 1A). Ninety-nine percent (521 of 524) of patients with colon cancer had genetic alterations or transcriptional reprogramming in at least one of these eight alleles, showing that these are highly conserved changes in human disease (Fig. 1B). By crossing these eight alleles, six genetically engineered colon models in three broad categories were generated, as described previously (26, 31, 32): tumors driven by APC loss, oncogenic KRAS activation, or oncogenic BRAF activation (Fig. 1C). We compared each of these six mouse models to the clinical classification system, CMS of colon cancer (4) by gene expression profiling of the primary tumors of each mouse genotype and used the CMScaller package (33) to correlate gene expression signatures with clinical subtypes. Each model had a unique CMS profile (Fig. 1D). *Kras^G12D/+^ Trp53^fl/fl^ Rosa26^N1icd/+^* (KPN) and *Apc^fl/+^ Kras^G12D/+^ Trp53^fl/fl^ Tgfbr1^fl/fl^* (AKPT) mice were closely related to the human CMS4 subtype, and the other models were more closely correlated with CMS2 and CM3 subtypes (Fig. 1D). *Apc^fl/+^ Kras^G12D/+^ Trp53^fl/fl^Rosa26^N1icd/+^* (AKPN) had a CMS1, CMS2, and CMS3 profile, whereas APC loss alone (*Apc^fl/+^*) was the least CMS1-like (Fig. 1D). These data show at least some subtype diversity between the colon cancer models, particularly between CMS2/3 and CMS4. To identify different phenotypic features, we also correlated gene set enrichment scores to the cancer hallmark gene sets using gene set variation analysis (GSVA; ref. 34). Each colon cancer model showed diverse hallmarks (Fig. 1E; Supplementary Fig. S1). There were similarities between AKPT and KPN, which had high scores for IL6/JAK/STAT signaling and IFN response. *Apc^fl/+^ Kras^G12D/+^* (AK) was high for mTORC1 signaling, E2F, G2M checkpoint, Myc, and glycolysis, suggesting high proliferation. *Braf^V600E/+^ Mlh1^fl/fl^ Tgfbr2^fl/fl^* (BMT), AKPN, AK, and *Apc^fl/+^* (A) had high fatty acid metabolism and oxidative phosphorylation profiles (Fig. 1E; Supplementary Fig. S1). Together, these data demonstrate the molecular and genetic diversity of these colon cancer models. This diversity suggests that these model systems would be useful for the systematic investigation of the genetic and transcriptional determinants of multitracer PET phenotypes across the breadth of colon cancer.

### Distinct intermodel heterogeneity in PET imaging signatures

**Figure 2.**
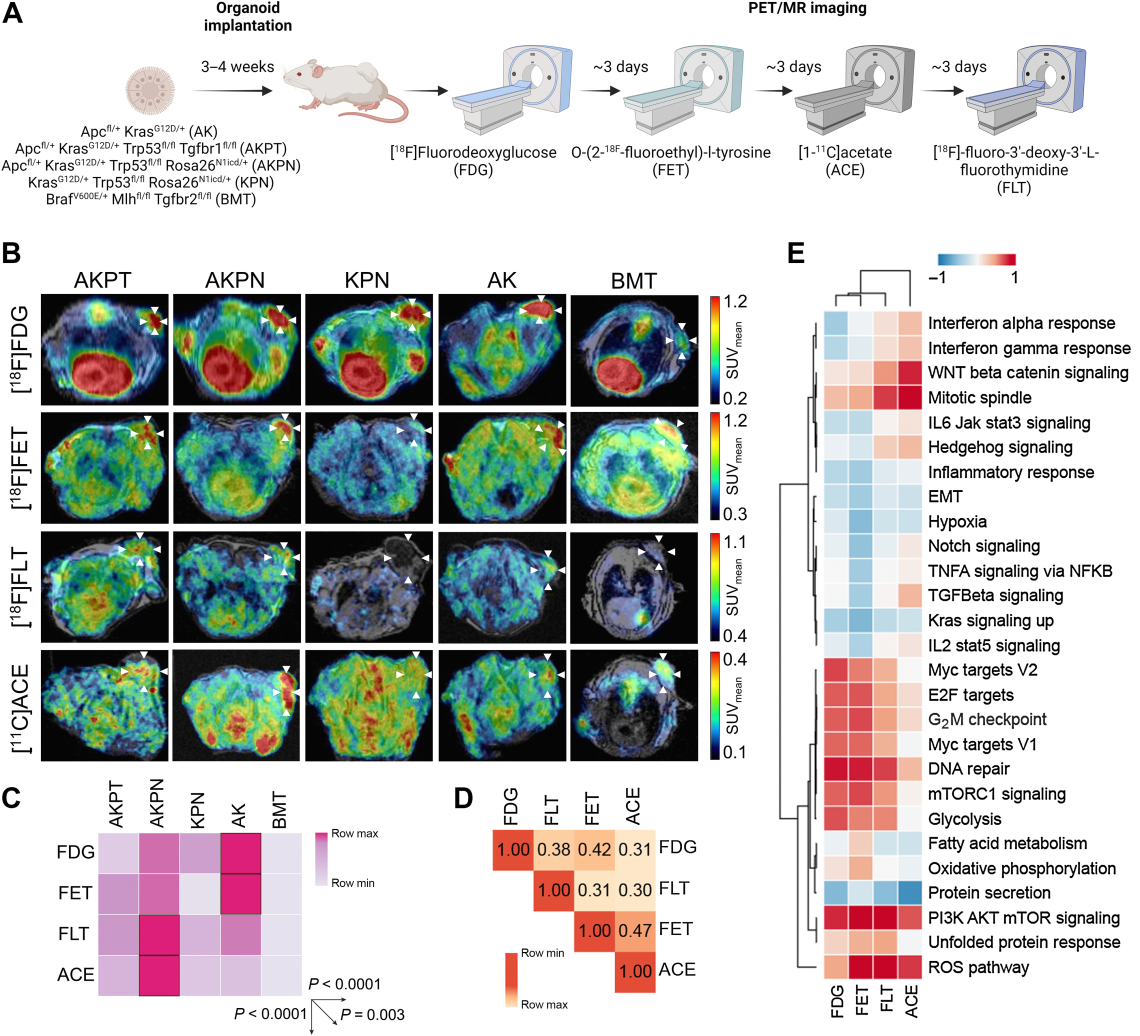
Distinct intermodel heterogeneity in PET imaging signatures. **A,** In the experimental imaging protocol, five colon cancer organoid models and four PET tracers were used to determine imaging signatures. Details of all mice used in these studies are presented in Supplementary Table S1. **B,** Representative transverse PET images from each model and tracer. The [^18^F]FDG PET/MR images of the KPN subcutaneous model are reproduced again in Figs. 4B and D and 5B for comparison against other tumors at different sites and stages. **C,** Imaging signature heatmap showing mean tracer uptake, models with highest tracer update highlighted with black outline (representation of the data matrix analyzed with two-way ANOVA). **D,** Correlation matrix of each tracer uptake based on Pearson correlation coefficient. **E,** Heatmap illustrating correlation of PET tracer uptake with gene expression in the Molecular Signatures Database (MSigDB) hallmark dataset, sorted by hierarchal clustering. (**A**, Created with BioRender.com.)

We aimed to establish whether PET imaging phenotypes varied among different colon cancer models with distinct tumor genetics and molecular subtypes. We investigated the intermodal heterogeneity in PET imaging phenotypes. To facilitate high-throughput imaging, we utilized organoids from five GEMM (AK, BMT, AKPT, AKPN, KPN), and subcutaneously implanted them into recipient mice (Supplementary Fig. S2; refs. 26, 31, 32). After 2 weeks of tumor growth, each mouse underwent four scans with different PET radiotracers, namely glucose uptake ([^18^F]FDG), amino acid uptake ([^18^F]FET), proliferation ([^18^F]FLT), and fatty acid synthesis ([^11^C]acetate; ACE; refs. 12, 39, 40; Fig. 2A). Notably, we observed significant intermodel heterogeneity in imaging signatures [F(4, 76) = 7.297; *P* < 0.0001], with distinct variations in the uptake of each tracer [F(3, 76) = 92.31; *P* < 0.0001] and differing uptake patterns depending on the model and tracer [F(12, 76) = 2.795; *P* = 0.003], thus indicating a unique tracer uptake profile in each model (Fig. 2B and C). Principal component analysis (PCA) revealed distinct and overlapping clusters corresponding to the different subcutaneous models (Supplementary Fig. S3). The AK organoid subcutaneous model exhibited the highest uptake of [^18^F]FDG and [^18^F]FET, whereas the AKPN subcutaneous model demonstrated the highest uptake of [^18^F]FLT and [^11^C]ACE (Fig. 2B and C). Notably, the KPN subcutaneous model exhibited low uptake of [^18^F]FET, whereas the AKPT subcutaneous model showed low uptake of [^18^F]FDG, and the BMT subcutaneous model consistently displayed the lowest uptake among all tracers. To determine if different PET radiotracers provided unique information, we correlated tracer uptake in each mouse and model and found only a limited correlation (*R*^2^ range = 0.30–0.47; Fig. 2D). In the PCA biplot, the radiotracer loadings were orthogonal, particularly for [^18^F]FET and [^18^F]FLT (Supplementary Fig. S3), suggesting that the variation in uptake of one tracer could not be fully explained by the uptake of another. Finally, to establish a link between PET imaging and molecular data, we correlated the uptake of each tracer in each subcutaneous model with the hallmarks of cancer pathways derived from transcriptional profiling performed in the GEMM of the same genotype (Fig. 2E). As anticipated, glycolysis was most strongly correlated with [^18^F]FDG uptake (*R* = 0.66), but overall [^18^F]FDG was highly associated with DNA repair (*R* = 0.81), [^18^F]FET with the ROS pathway (*R* = 0.97), [^18^F]FLT with PI3k Akt mTOR signaling (*R* = 0.95). Surprisingly, [^11^C]ACE uptake was linked to mitotic spindle (*R* = 0.89) and Wnt/βcatenin signaling (*R* = 0.77) but not fatty acid metabolism (*R* = −0.21). The high content of short-chain fatty acids in the colonic environment, produced by the microbiome, might be reducing *de novo* fatty acid synthesis (41). Taken together, these findings indicate that this panel of PET tracers is complementary and is associated with distinct molecular and genetic features in these colon cancer models.

### PET imaging can distinguish different colon cancer models and individual driver genes

**Figure 3.**
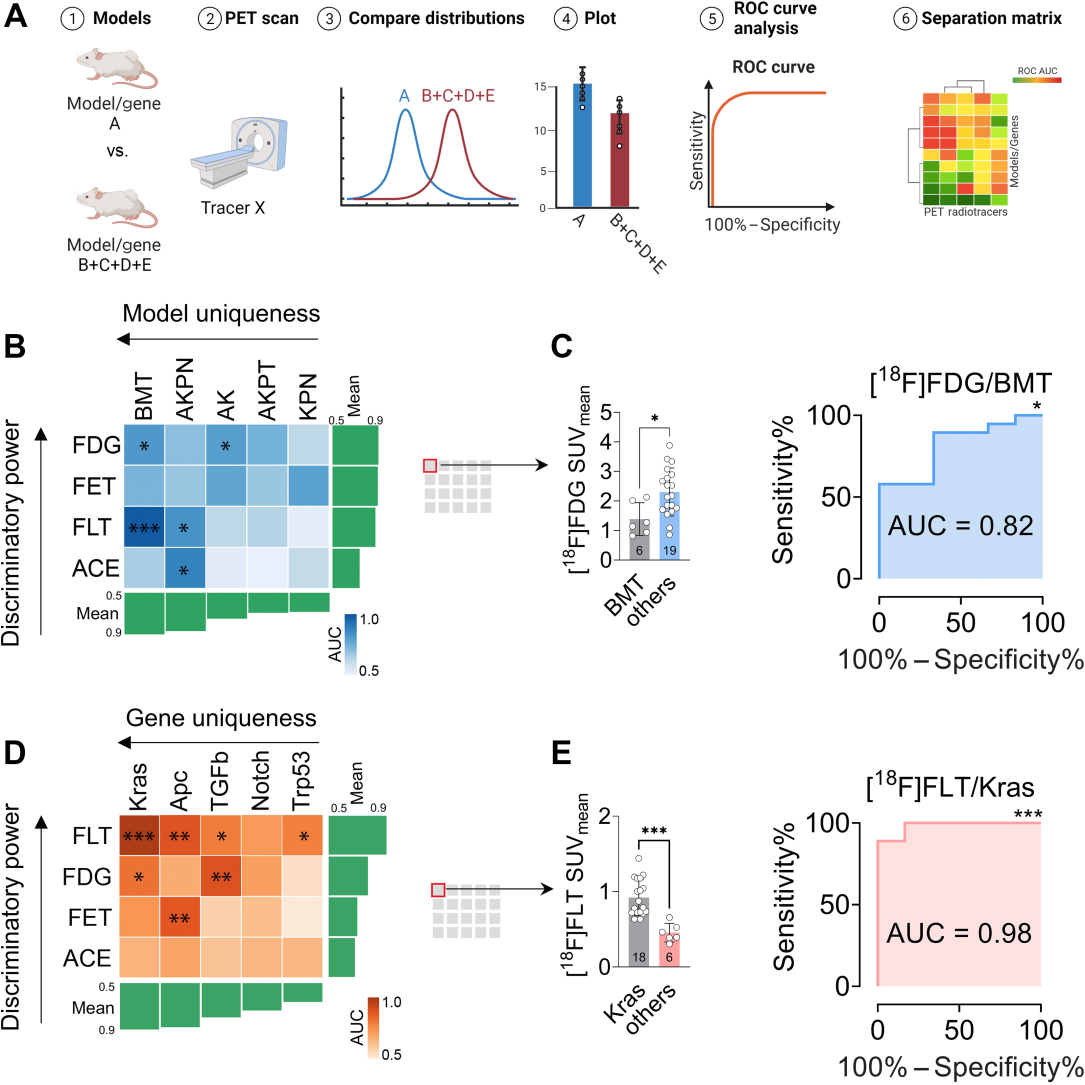
PET imaging can distinguish different colon subcutaneous organoid cancer models and individual driver genes. **A,** The data processing workflow for comparing PET radiotracer discriminatory power and the model/gene uniqueness. **B,** Separation matrix and statistics of the area under the ROC curves for each tracer and model. **C,** Red highlighted box showing boxplot and ROC curves for [^18^F]FDG in the BMT (*n* = 6 subcutaneous organoid allografts) compared with other models (*n* = 19), each point represents a mouse. Numbers inside bars show sample size, *n*. Data compared using unpaired *t* test. **D,** Separation matrix and statistics of the area under the ROC curves for each tracer and gene. *Tgfbr1/Alk5 ^fl/fl^* and *Tgfbr2 ^fl/fl^* are combined as TGFb for this analysis. **E,** Red highlighted box showing boxplot and ROC curves for [^18^F]FLT in the Kras (*n* = 18) compared to other subcutaneous models (*n* = 6), each point represents a mouse. Numbers inside bars show n. Data compared using unpaired *t* test. Error bars in **C** and **D** represent SD. *, *P* < 0.05; **, *P* < 0.01; ***, *P* < 0.001 for unpaired *t* tests and AUC ROC. Each analysis stands on its own; no multiple comparison testing was used. See extended datasets in Supplementary Fig. S4. (**A**, Created with BioRender.com.)

To investigate the capacity of each PET tracer ([^18^F]FDG, [^18^F]FET, [^18^F]FLT, and [^11^C]ACE) to distinguish each colon organoid subcutaneous model (BMT, AKPN, AK, AKPT, KPN), we analyzed the areas under the receiver-operating characteristic (ROC) curve for each radiotracer and model. The area under the curve (AUC) provides the overall performance of each PET tracer as a binary classifier across all thresholds (Fig. 3A). To visualize the results, we generated a separation matrix for each PET radiotracer and colon model, ranking the mean AUC values from highest to lowest to assess the overall PET uniqueness of each model. On the basis of the uptake of the four PET radiotracers tested, the BMT subcutaneous model was the most distinct (ROC_mean_ = 0.80), followed by the AKPN (ROC_mean_ = 0.77), AK (ROC_mean_ = 0.68), AKPT (ROC_mean_ = 0.64), and KPN subcutaneous models (ROC_mean_ = 0.64; Fig. 3B). We also assessed the discriminatory power of each PET radiotracer by comparing the mean AUC for each ROC curve: [^18^F]FDG (ROC_mean_ = 0.73) > [^18^F]FET (ROC_mean_ = 0.73) > [^18^F]FLT (ROC_mean_ = 0.72) > [^11^C]ACE (ROC_mean_ = 0.64). This indicated that [^18^F]FDG and [^18^F]FET performed the best overall when separating models, closely followed by [^18^F]FLT (Fig. 3B). Interestingly, different PET tracers were more effective in discriminating different models. The most effective separators for each tracer and model were [^18^F]FDG/BMT, [^18^F]FET/KPN, [^18^F]FLT/BMT, and [^11^C]ACE/AKPN with AUC values of 0.82 ± 0.09 (*P* = 0.02), 0.79 ± 0.09 (*P* = 0.05), 0.98 ± 0.02 (*P* < 0.001), and 0.88 ± 0.08 (*P* = 0.02), respectively (Fig. 3B and C; Supplementary Figs. S4A and S4B).

Previous studies suggest that specific oncogenes have distinct phenotypic features, such as KRAS driving a glycolytic phenotype (12, 42, 43). However the extent to which other tumor drivers contribute to PET imaging phenotypes remains unclear. To determine whether a particular oncogene or tumor suppressor produces an imaging phenotype independently of other alterations, we grouped and compared models based on their genetics (Fig. 3D and E; Supplementary Figs. S4 and S5). The separation matrix revealed that overall, the *Kras* mutation (ROC_mean_ = 0.80) had the most distinctive driver phenotype, followed by *Apc* (ROC_mean_ = 0.78), *Tgfbr1* (ROC_mean_ = 0.72), and *Notch* (ROC_mean_ = 0.67). The least distinguishable genetic alteration was the loss of the tumor suppressor p53. When separating the driver genes, [^18^F]FLT exhibited the best performance, followed by [^18^F]FDG, [^18^F]FET, and [^11^C]ACE. ROC curves were used to determine the optimal cut-off values for maximizing separation. The optimal separator overall was [^18^F]FLT SUV_mean_ 0.68, which had 88.9% sensitivity and 100% specificity for distinguishing *Kras* mutated tumors from other genetic alterations. These data demonstrate that a collection of colon cancer models with different genetic and phenotypic characteristics can be noninvasively distinguished using PET imaging. Some models and genes, particularly *Kras* and *Apc*, were more distinctive than others and tracers beyond [^18^F]FDG, such as [^18^F]FET and [^18^F]FLT proved valuable in distinguishing between models and genetics. Finally, to explore if imaging differences based on transcriptional profiles were driven by CMS, we divided the models into two classes, CM4 and CMS2/3, and performed ROC analysis. However, none of the tested radiotracers were able to differentiate based on the CMS classifiers (Supplementary Figs. S5 and S6).

### Imaging signatures depend on tumor context as well as tumor genetics

**Figure 4.**
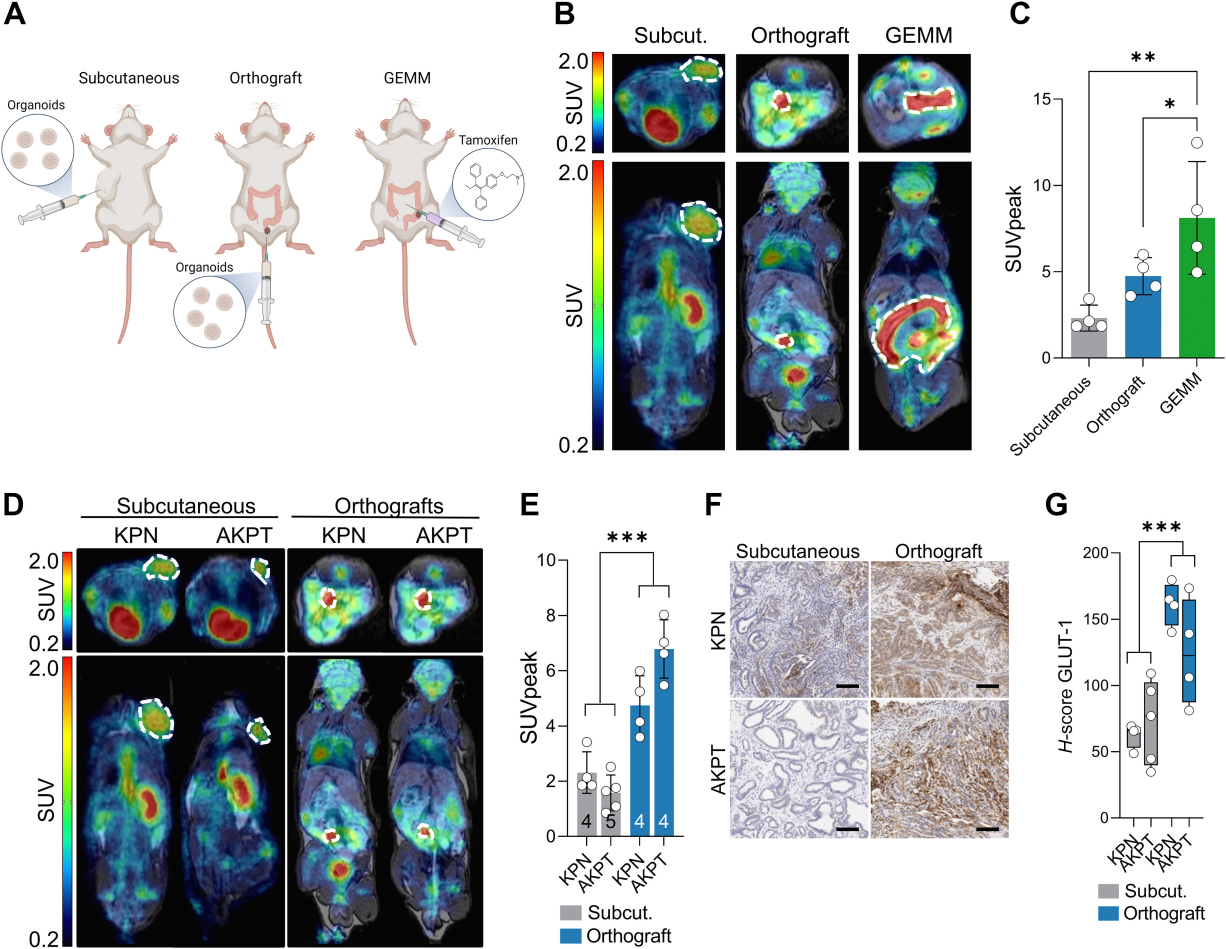
Imaging signatures depend on tumor context. **A,** The generation of mouse models. **B**, Transverse and coronal PET/MRI slices images showing [^18^F]FDG uptake in subcutaneous and orthotopic organoid models and GEMM of *Kras^G12D/+^ Trp53^fl/fl^ Rosa26^N1icd/+^* (KPN) colon cancer. KPN subcutaneous images reproduced here from Fig. 2B for comparison with KPN orthograft and GEMM. Tumors are outlined with a white dotted line. **C,** Standard uptake peak values (SUV_peak_) PET quantification from images in **B** (*n* = 4 mice/model). Data compared using ANOVA followed by Fisher least significant difference test. **D,** Transverse and coronal PET/MRI slices images showing [^18^F]FDG uptake in subcutaneous and orthotopic KPN and *Apc^fl/+^ Kras^G12D/+^ Trp53^fl/fl^ Tgfbr1 ^fl/fl^* (AKPT) organoid models of colon cancer. Tumors are outlined with a white dotted line. KPN images reproduced here from Figs. 2B and 4B and for comparison to AKPT. **E,** SUV_peak_ quantification from images in panel **D** (Numbers inside bars show sample size, n). Data compared using two-way ANOVA, with the results of injection site shown. Details of all mice used in these studies are presented in Supplementary Table S1. **F,** Representative GLUT-1 immunohistochemistry of tumors from mice shown in panel **E**. Black scale bars represents 100 μm. **G,** H-score of GLUT-1 immunohistochemistry from mice shown in panel **E**. Box and whisker plots show range, median and interquartile range. Error bars in panel **C** and **E** represent standard deviation. Data compared using two-way ANOVA, with the results of injection site shown, * *P* < 0.05, ** *P* < 0.01, *** *P* < 0.001. (**A**, Created with BioRender.com.)

In addition to tumor genotype, the tumor environment is an important driver of tumor phenotype and, consequently, the imaging phenotype (44, 45). Therefore, we sought to determine the relative contributions of genes and the environment to imaging signatures. To investigate the influence of the tissue environment, we utilized the same genetic model (KPN) in different contexts: subcutaneous implantation, orthotopic (intracolonic implantation), or autochthonously driven directly from conditionally expressed alleles (i.e., GEMM; Fig. 4A). The subcutaneously implanted mice are the same mice as used for analysis in Figs. 2 and 3. We monitored orthotopic tumor growth by colonoscopy (Supplementary Fig. S7). We observed that higher-fidelity models had greater [^18^F]FDG uptake, GEMM > orthotopic > subcutaneous (*F* = 8.2; *P* = 0.009), suggesting an environmental effect due to tumor location on the PET imaging phenotype (Fig. 4B and C). To determine the relative effect of genes and environment, we compared KPN and AKPT subcutaneously and orthotopically, and performed PET imaging using [^18^F]FDG. Although [^18^F]FDG uptake in orthografts was higher than in subcutaneous tumors (74.2% variation; *P* < 0.0001), the extent of this increase varied by genotype (KPN vs. AKPT; 9.8% variation; *P* = 0.0067; Fig. 4D and E). To validate these findings, we stained subcutaneous and orthotopic tissues for GLUT-1 (SLC2A1) and showed higher (*P* < 0.001) GLUT-1 transporter expression in orthografts (Fig. 4F and G), implying higher glucose uptake. These data suggest that the tumor environment is a major determinant of imaging phenotype. Moreover, the relative contribution of genes and the environment to imaging signatures was partially dependent on tumor genetics, revealing a previously unreported interaction between genes and environment in shaping the PET imaging phenotype.

### PET imaging phenotypes change with cancer progression

Cancer cells can undergo evolutionary changes over time, acquiring aggressive features that enable extravasation, circulation, homing, and colonization of distant sites. PET staging is a crucial application of this technology, facilitating the identification of locally advanced and metastatic disease. Although [^18^F]FDG has been extensively used for this purpose, the potential of other tracers to identify metabolic progression remains largely unexplored. Given the significant influence of genes and the environment on PET signatures, our final objective was to assess the relative impact of the tumor metastatic process on imaging signatures using a range of PET tracers.

**Figure 5.**
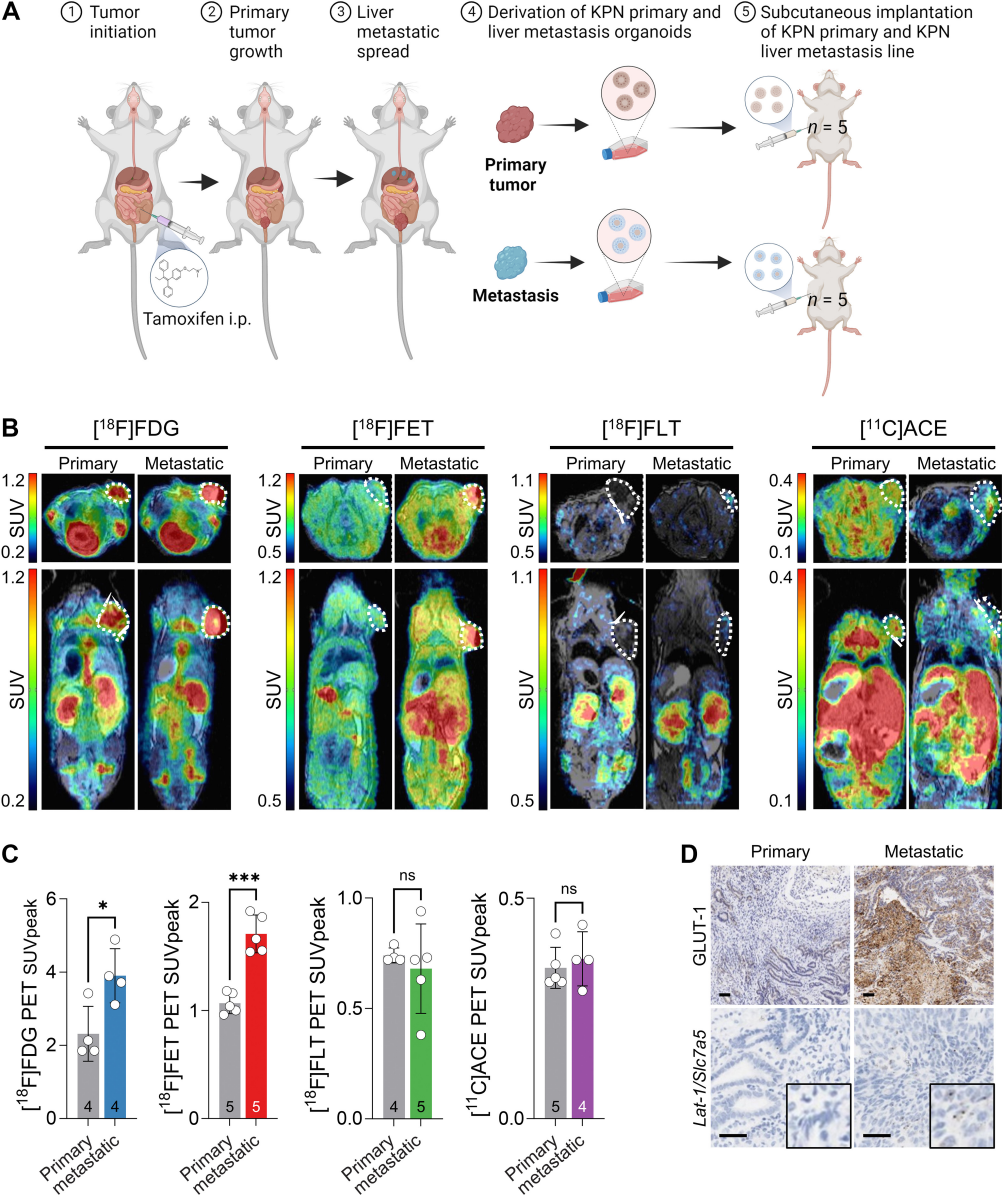
PET imaging phenotypic difference between primary and metastatic tumors. **A,** The generation of the *Kras^G12D/+^ Trp53^fl/fl^ Rosa26^N1icd/+^* (KPN) and KPN liver metastasis organoid lines and subsequent implantation. One pair of lines, generated from a matched mouse primary tumor and liver metastasis, which were then propagated and injected subcutaneously into recipient mice (*n* = 5). **B,** Transverse and coronal PET/MRI slice images showing uptake of four PET tracers ([^18^F]FDG, [^18^F]FET, [^18^F]FLT, [^18^F]ACE) in subcutaneously implanted KPN primary and KPN liver metastasis organoids. KPN primary tumor-bearing mice are the same four PET ([^18^F]FDG, [^18^F]FET, [^18^F]FLT, [^18^F]ACE) images (primary) as displayed in Figs. 2B and 4B and D. **C,** Standard uptake peak values (SUV_peak_) PET quantification from images in **B**. Sample size (*n*) is displayed on the bars. Error bars represent SD. Data compared using unpaired *t* test. Details of all mice used in these studies are presented in Supplementary Table S1. **D,** Representative GLUT-1 IHC and *Lat-1/Slc7a5* ISH. Black scale bars represent 50 μm (*, *P* < 0.05; ***, *P* < 0.001; see also Supplementary Fig. S8). (**A**, Created with BioRender.com.)

We isolated tumors and cultured organoids from a matched primary and liver metastasis obtained from a KPN mouse, designated KPN (primary) and KPN (metastatic), respectively (Fig. 5A). As tumor environment had an influence, we implanted both primary and metastatic cells in the subcutaneous site to isolate the specific changes in PET signatures associated with metastatic progression. Following tumor growth, we performed multitracer PET imaging (Fig. 5B). The KPN primary imaging data are the same as presented in Fig. 2. The levels of [^18^F]FDG (*P* = 0.028) and [^18^F]FET (*P* = 0.008) were found to be higher in the metastatic model, whereas [^11^C]ACE and [^18^F]FLT did not show significant differences (Fig. 5C). These data were further supported by increased staining of GLUT-1 (*P* < 0.0001) and *Lat-1/Slc7a5* expression (*P* = 0.037) in the metastatic model compared with the primary tumor model (Fig. 5D; Supplementary Fig. S8), indicating higher glucose and amino acid uptake detectable by PET imaging during cancer progression in this primary and metastatic pair.

## Discussion

The primary objective of our study was to investigate the contributing factors, including genotype, tumor environment, and stage, that influence the imaging phenotypes observed in multitracer PET imaging of colon cancer. To accomplish this, we utilized a diverse panel of colon cancer GEMM to discern the genotypes in primary mouse organoid models. Our results suggest distinct imaging phenotypes in models with a gain of oncogenic Kras and loss of Apc, TGFβ, or p53. Specifically, we observed that high uptake of [^18^F]FLT and [^18^F]FDG was associated with Kras mutation, whereas low uptake was linked to TGFβ loss. Moreover, loss of Apc was associated with elevated uptake of [^18^F]FET or [^18^F]FLT. Notably, the separation between Kras and Braf genotypes was the most pronounced, confirming the dominant role of Kras in determining metabolic phenotype. These findings support previous studies that have associated higher [^18^F]FDG uptake with Kras-mutated colorectal tumors (17, 22, 43), but the association of high [^18^F]FLT with Kras-mutated tumors has not been reported previously (16). Furthermore, our study demonstrated higher uptake of [^18^F]FET (*P* = 0.004) and [^18^F]FLT (*P* < 0.001) in models with Apc loss, which aligns with our recent data indicating that intestinal Apc and Kras mutations enhance [^18^F]FET uptake through SLC7A5/LAT-1, resulting in increased protein synthesis (32). These findings suggest that [^18^F]FET may have a role in distinguishing tumors with Apc loss. Overall, our results indicate that the PET tracers utilized in this study may hold clinical utility for noninvasive genetic profiling and subsequent patient selection. Although FDG has struggled to accurately differentiate Kras due to poor specificity (22), the potential of [^18^F]FLT and [^18^F]FET in distinguishing Kras and Apc loss respectively warrants further investigation, as their ROC AUC values exceeded those of [^18^F]FDG ([^18^F]FLT/Kras 0.98 and [^18^F]FET/Apc 0.89 vs. [^18^F]FDG/Kras 0.82). Although these findings hold promise for clinical applications, further validation is necessary to establish their clinical significance.

Our study also uncovered that the tissue environment exerts a greater influence on PET imaging signatures than cancer genetics alone. To explore this, we implanted the same genetic organoid lines in different tissue environments, either subcutaneously or in the colon submucosa. Remarkably, we found that changing the implantation site had a substantially larger impact (74.2%) on the [^18^F]FDG phenotype compared with alterations in genes such as Apc and TGFβ loss or Notch gain (2.2%). It is widely known that the tumor environment via altered perfusion, hypoxia, and alterations in metabolic pathways and transporters, plays a crucial role in determining the metabolic phenotype and may be a key driver of the observed PET differences in our study (44–46). The colon possesses a unique nutritional environment with varying levels of amino acids, sugars, and acetate, which could be relevant to the metabolic phenotypes observed in colon cancer (47). Although we were unable to measure all models intracolonically due to resource constraints, our findings indicate that cell extrinsic mechanisms play a significant role in imaging phenotypes. Moreover, our study suggests that PET imaging can identify adaptive changes in cancer cells resulting from exposure to new nutritional environments. It is important to consider these environmental differences when using PET for stratification based on tumor genetics, as posttreatment modification of the tumor microenvironment through immunotherapy, radiotherapy, or surgery could complicate PET-based stratification.

In the metastatic setting, our findings demonstrated that even after accounting for the impact of the tumor environment, a metastatic tumor exhibited elevated glucose uptake, consistent with previous reports of increased glycolysis in liver metastases (48). This elevated glycolysis may play a crucial role in metabolic plasticity, supporting invasion, migration, and potentially epithelial-to-mesenchymal transition (EMT) during cancer progression (42). In addition, we observed increased uptake of amino acids with [^18^F]FET, which targets SLC7a5/LAT-1, a critical transporter for maintaining intracellular amino acid pools necessary for cancer cell dissemination. Notably, deletion of SLC7a5/LAT-1 has been shown to reduce metastasis and prolong survival in the KPN model (32). These findings suggest that PET imaging with [^18^F]FET and [^18^F]FDG can identify aggressive features acquired during cancer progression and the metastatic cascade, however heterogeneity in KPN tumors means that the imaging changes observed here are unlikely to be universal features of metastasis (32, 49). [^18^F]FET or other amino acid radiotracers such as [^11^C]glutamine may serve as valuable additions for tumor staging (50).

Our study had several limitations. Although we employed a diverse panel of tracers and models, they were not exhaustive. Clinical subgroups, such as CMS1, were underrepresented, and the separation between the CMS2 and CMS3 subtypes was limited. Addressing these limitations would require further expansion of the models and incorporation of datasets from both mice and humans to improve stratification methods (51). We did not observe distinct PET imaging phenotypes between CMS2/3 and CMS4 subtypes. The ability to discriminate among these subtypes was dependent on the panel of PET radiotracers, which were not exhaustive, particularly focusing on metabolism. Including PET tracers targeting stromal components, such as fibroblasts (^68^Ga-FAPI; ref. 52) or immune cells (^89^ZED88082A; ref. 11), may enhance discriminatory power and enable the distinction of tumor populations, particularly CMS1 and CMS4 subtypes (11). In addition, we observed phenotypic heterogeneity within CMS classes, as evidenced by variations in cancer hallmarks even within seemingly similar CMS groups, such as AK and AKPN, both of which are classified as CMS2/3 but exhibit different cancer hallmarks. Future imaging-based classification approaches may need to adopt alternative strategies, such as addressing phenotypic variations rather than relying solely on translational classifiers. Furthermore, extending the experiments to clinical material, including the addition of patient-derived xenografts, would help validate whether similar effects are observed in more complex genetic scenarios. It is important to acknowledge that determining the sensitivity and specificity of these tracers in a clinical context would require prospective studies, and certain tracers, such as [^18^F]FLT, exhibit high background uptake in areas of frequent colorectal metastasis, such as the liver.

Although our findings hold promise for altering therapeutic pathways, further validation is necessary before implementing them in clinical practice. However we can speculate that patients that have high [^18^F]FET and [^18^F]FLT are more likely to have Apc loss and could be susceptible to drug targeting of the Wnt signaling pathway. Similarly, patients with high [^18^F]FDG and [^18^F]FLT are more likely to have Kras mutations and therefore poor response to EGFR inhibition (22). Conversely, low [^18^F]FDG and [^18^F]FLT suggests alterations in TGFβ signaling, and these patients may have improved response to MEK or EGFR inhibitors (53). Ultimately, to establish the clinical significance of our findings, it is crucial to conduct further studies in patient cohorts, incorporating prospective multitracer biomarker validation.

In summary, our study demonstrated that the imaging phenotypes of colon cancer are influenced by factors such as genotype, model, site, and stage, indicating that they arise from a combination of intrinsic and extrinsic tumor mechanisms. These findings support the use of PET as a valuable biological imaging modality that provides unique molecular diagnostic information. We achieved high accuracy in distinguishing models and noninvasively differentiating cancer genetics, even in the presence of complex driver genes. The tumor environment emerged as a key driver of imaging phenotypes, surpassing genetic differences in certain scenarios. Radiotracers like [^18^F]FLT and [^18^F]FET warrant further investigation in the assessment of *Kras* mutation, *Apc* loss, and tumor staging. PET-based noninvasive phenotyping offers a complementary approach to current biopsy-based molecular diagnosis and provides the opportunity to assess the entire body, identify intrapatient heterogeneity, and monitor dynamic changes. This study represents a step toward noninvasive, multitracer profiling of cancer subtypes, with the potential to inform precision medicine approaches in the field of colon cancer.

## Supplementary Material

Table S1 Models Overview

Supplementary Data 1 Supplementary Figures S1-S8

## References

[1] Siegel RL , Wagle NS, Cercek A, Smith RA, Jemal A. Colorectal cancer statistics, 2023. CA Cancer J Clin 2023;73:233–54. 36856579 10.3322/caac.21772

[2] Dunne PD , McArt DG, Bradley CA, O'Reilly PG, Barrett HL, Cummins R, . Challenging the cancer molecular stratification dogma: intratumoral heterogeneity undermines consensus molecular subtypes and potential diagnostic value in colorectal cancer. Clin Cancer Res 2016;22:4095–104. 27151745 10.1158/1078-0432.CCR-16-0032

[3] Eide PW , Moosavi SH, Eilertsen IA, Brunsell TH, Langerud J, Berg KCG, . Metastatic heterogeneity of the consensus molecular subtypes of colorectal cancer. NPJ Genom Med 2021;6:59. 34262039 10.1038/s41525-021-00223-7 PMC8280229

[4] Guinney J , Dienstmann R, Wang X, de Reynies A, Schlicker A, Soneson C, . The consensus molecular subtypes of colorectal cancer. Nat Med 2015;21:1350–6. 26457759 10.1038/nm.3967 PMC4636487

[5] Xie YH , Chen YX, Fang JY. Comprehensive review of targeted therapy for colorectal cancer. Signal Transduct Target Ther 2020;5:22. 32296018 10.1038/s41392-020-0116-z PMC7082344

[6] Ten Hoorn S , de Back TR, Sommeijer DW, Vermeulen L. Clinical value of consensus molecular subtypes in colorectal cancer: a systematic review and meta-analysis. J Natl Cancer Inst 2022;114:503–16. 34077519 10.1093/jnci/djab106 PMC9002278

[7] Alieva M , Margarido AS, Wieles T, Abels ER, Colak B, Boquetale C, . Preventing inflammation inhibits biopsy-mediated changes in tumor cell behavior. Sci Rep 2017;7:7529. 28790339 10.1038/s41598-017-07660-4 PMC5548904

[8] Backes Y , Seerden TCJ, van Gestel R, Kranenburg O, Ubink I, Schiffelers RM, . Tumor seeding during colonoscopy as a possible cause for metachronous colorectal cancer. Gastroenterology 2019;157:1222–32. 31419435 10.1053/j.gastro.2019.07.062

[9] Hobson J , Gummadidala P, Silverstrim B, Grier D, Bunn J, James T, . Acute inflammation induced by the biopsy of mouse mammary tumors promotes the development of metastasis. Breast Cancer Res Treat 2013;139:391–401. 23715631 10.1007/s10549-013-2575-1 PMC4038002

[10] Alderdice M , Richman SD, Gollins S, Stewart JP, Hurt C, Adams R, . Prospective patient stratification into robust cancer-cell intrinsic subtypes from colorectal cancer biopsies. J Pathol 2018;245:19–28. 29412457 10.1002/path.5051 PMC5947827

[11] Kist de Ruijter L , van de Donk PP, Hooiveld-Noeken JS, Giesen D, Elias SG, Lub-de Hooge MN, . Whole-body CD8(+) T cell visualization before and during cancer immunotherapy: a phase 1/2 trial. Nat Med 2022;28:2601–10. 36471036 10.1038/s41591-022-02084-8 PMC9800278

[12] Lewis DY , Soloviev D, Brindle KM. Imaging tumor metabolism using positron emission tomography. Cancer J 2015;21:129–36. 25815854 10.1097/PPO.0000000000000105 PMC4413031

[13] Bensch F , van der Veen EL, Lub-de Hooge MN, Jorritsma-Smit A, Boellaard R, Kok IC, . ^89^Zr-atezolizumab imaging as a non-invasive approach to assess clinical response to PD-L1 blockade in cancer. Nat Med 2018;24:1852–8. 30478423 10.1038/s41591-018-0255-8

[14] van Kruchten M , de Vries EGE, Brown M, de Vries EFJ, Glaudemans A, Dierckx R, . PET imaging of oestrogen receptors in patients with breast cancer. Lancet Oncol 2013;14:e465–e75. 24079874 10.1016/S1470-2045(13)70292-4

[15] Cherry SR , Jones T, Karp JS, Qi J, Moses WW, Badawi RD. Total-Body PET: maximizing sensitivity to create new opportunities for clinical research and patient care. J Nucl Med 2018;59:3–12. 28935835 10.2967/jnumed.116.184028 PMC5750522

[16] McKinley ET , Ayers GD, Smith RA, Saleh SA, Zhao P, Washington MK, . Limits of [^18^F]-FLT PET as a biomarker of proliferation in oncology. PLoS One 2013;8:e58938. 23554961 10.1371/journal.pone.0058938 PMC3598948

[17] Iwamoto M , Kawada K, Nakamoto Y, Itatani Y, Inamoto S, Toda K, . Regulation of ^18^F-FDG accumulation in colorectal cancer cells with mutated KRAS. J Nucl Med 2014;55:2038–44. 25453050 10.2967/jnumed.114.142927

[18] Lee ST , Tebbutt N, Gan HK, Liu Z, Sachinidis J, Pathmaraj K, . Evaluation of ^18^F-FMISO PET and (18)F-FDG PET scans in assessing the therapeutic response of patients with metastatic colorectal cancer treated with anti-angiogenic therapy. Front Oncol 2021;11:606210. 33816239 10.3389/fonc.2021.606210 PMC8010243

[19] Hensley CT , Faubert B, Yuan Q, Lev-Cohain N, Jin E, Kim J, . Metabolic heterogeneity in human lung tumors. Cell 2016;164:681–94. 26853473 10.1016/j.cell.2015.12.034 PMC4752889

[20] Patel S , McCall M, Ohinmaa A, Bigam D, Dryden DM. Positron emission tomography/computed tomographic scans compared to computed tomographic scans for detecting colorectal liver metastases: a systematic review. Ann Surg 2011;253:666–71. 21475005 10.1097/SLA.0b013e31821110c9

[21] Chen SH , Miles K, Taylor SA, Ganeshan B, Rodriquez M, Fraioli F, . FDG-PET/CT in colorectal cancer: potential for vascular-metabolic imaging to provide markers of prognosis. Eur J Nucl Med Mol Imaging 2021;49:371–84. 33837843 10.1007/s00259-021-05318-y PMC8712298

[22] Kawada K , Toda K, Nakamoto Y, Iwamoto M, Hatano E, Chen F, . Relationship between ^18^F-FDG PET/CT scans and KRAS mutations in metastatic colorectal cancer. J Nucl Med 2015;56:1322–7. 26135109 10.2967/jnumed.115.160614

[23] Shibata H , Toyama K, Shioya H, Ito M, Hirota M, Hasegawa S, . Rapid colorectal adenoma formation initiated by conditional targeting of the Apc gene. Science 1997;278:120–3. 9311916 10.1126/science.278.5335.120

[24] Jackson EL , Willis N, Mercer K, Bronson RT, Crowley D, Montoya R, . Analysis of lung tumor initiation and progression using conditional expression of oncogenic K-ras. Genes Dev 2001;15:3243–8. 11751630 10.1101/gad.943001 PMC312845

[25] Murtaugh LC , Stanger BZ, Kwan KM, Melton DA. Notch signaling controls multiple steps of pancreatic differentiation. Proc Natl Acad Sci USA 2003;100:14920–5. 14657333 10.1073/pnas.2436557100 PMC299853

[26] Leach JDG , Vlahov N, Tsantoulis P, Ridgway RA, Flanagan DJ, Gilroy K, . Oncogenic BRAF, unrestrained by TGFbeta-receptor signalling, drives right-sided colonic tumorigenesis. Nat Commun 2021;12:3464. 34103493 10.1038/s41467-021-23717-5 PMC8187652

[27] Mercer K , Giblett S, Green S, Lloyd D, DaRocha Dias S, Plumb M, . Expression of endogenous oncogenic V600EB-raf induces proliferation and developmental defects in mice and transformation of primary fibroblasts. Cancer Res 2005;65:11493–500. 16357158 10.1158/0008-5472.CAN-05-2211 PMC2640458

[28] Larsson J , Goumans MJ, Sjostrand LJ, van Rooijen MA, Ward D, Leveen P, . Abnormal angiogenesis but intact hematopoietic potential in TGF-beta type I receptor-deficient mice. EMBO J 2001;20:1663–73. 11285230 10.1093/emboj/20.7.1663 PMC145465

[29] Leveen P , Larsson J, Ehinger M, Cilio CM, Sundler M, Sjostrand LJ, . Induced disruption of the transforming growth factor beta type II receptor gene in mice causes a lethal inflammatory disorder that is transplantable. Blood 2002;100:560–8. 12091349 10.1182/blood.v100.2.560

[30] Jonkers J , Meuwissen R, van der Gulden H, Peterse H, van der Valk M, Berns A. Synergistic tumor suppressor activity of BRCA2 and p53 in a conditional mouse model for breast cancer. Nat Genet 2001;29:418–25. 11694875 10.1038/ng747

[31] Jackstadt R , van Hooff SR, Leach JD, Cortes-Lavaud X, Lohuis JO, Ridgway RA, . Epithelial NOTCH signaling rewires the tumor microenvironment of colorectal cancer to drive poor-prognosis subtypes and metastasis. Cancer Cell 2019;36:319–36. 31526760 10.1016/j.ccell.2019.08.003 PMC6853173

[32] Najumudeen AK , Ceteci F, Fey SK, Hamm G, Steven RT, Hall H, . The amino acid transporter SLC7A5 is required for efficient growth of KRAS-mutant colorectal cancer. Nat Genet 2021;53:16–26. 33414552 10.1038/s41588-020-00753-3

[33] Eide PW , Bruun J, Lothe RA, Sveen A. CMScaller: an R package for consensus molecular subtyping of colorectal cancer pre-clinical models. Sci Rep 2017;7:16618. 29192179 10.1038/s41598-017-16747-x PMC5709354

[34] Hanzelmann S , Castelo R, Guinney J. GSVA: gene set variation analysis for microarray and RNA-seq data. BMC Bioinf 2013;14:7. 10.1186/1471-2105-14-7 PMC3618321 23323831

[35] Hamacher K , Coenen HH. Efficient routine production of the 18F-labelled amino acid O-2–^18^F fluoroethyl-L-tyrosine. Appl Radiat Isot 2002;57:853–6. 12406628 10.1016/s0969-8043(02)00225-7

[36] Suehiro M , Vallabhajosula S, Goldsmith SJ, Ballon DJ. Investigation of the role of the base in the synthesis of [^18^F]FLT. Appl Radiat Isot 2007;65:1350–8. 17919915 10.1016/j.apradiso.2007.07.013

[37] Soloviev D , Tamburella C. Captive solvent [^11^C]acetate synthesis in GMP conditions. Appl Radiat Isot 2006;64:995–1000. 16806949 10.1016/j.apradiso.2006.04.011

[38] Hoadley KA , Yau C, Hinoue T, Wolf DM, Lazar AJ, Drill E, . Cell-of-origin patterns dominate the molecular classification of 10,000 tumors from 33 types of cancer. Cell 2018;173:291–304. 29625048 10.1016/j.cell.2018.03.022 PMC5957518

[39] Lewis DY , Boren J, Shaw GL, Bielik R, Ramos-Montoya A, Larkin TJ, . Late imaging with [1-^11^C]acetate improves detection of tumor fatty acid synthesis with PET. J Nucl Med 2014;55:1144–9. 24777291 10.2967/jnumed.113.134437

[40] Lewis DY , Mair R, Wright A, Allinson K, Lyons SK, Booth T, . [^18^F]fluoroethyltyrosine-induced cerenkov luminescence improves image-guided surgical resection of glioma. Theranostics 2018;8:3991–4002. 30083276 10.7150/thno.23709 PMC6071532

[41] Bae JM , Kim JH, Oh HJ, Park HE, Lee TH, Cho NY, . Downregulation of acetyl-CoA synthetase 2 is a metabolic hallmark of tumor progression and aggressiveness in colorectal carcinoma. Mod Pathol 2017;30:267–77. 27713423 10.1038/modpathol.2016.172

[42] Bergers G , Fendt SM. The metabolism of cancer cells during metastasis. Nat Rev Cancer 2021;21:162–80. 33462499 10.1038/s41568-020-00320-2 PMC8733955

[43] La Vecchia S , Sebastian C. Metabolic pathways regulating colorectal cancer initiation and progression. Semin Cell Dev Biol 2020;98:63–70. 31129171 10.1016/j.semcdb.2019.05.018

[44] Sullivan MR , Danai LV, Lewis CA, Chan SH, Gui DY, Kunchok T, . Quantification of microenvironmental metabolites in murine cancers reveals determinants of tumor nutrient availability. eLife 2019;8:e44235. 30990168 10.7554/eLife.44235 PMC6510537

[45] Davidson SM , Papagiannakopoulos T, Olenchock BA, Heyman JE, Keibler MA, Luengo A, . Environment impacts the metabolic dependencies of ras-driven non-small cell lung cancer. Cell Metab 2016;23:517–28. 26853747 10.1016/j.cmet.2016.01.007 PMC4785096

[46] Vande Voorde J , Ackermann T, Pfetzer N, Sumpton D, Mackay G, Kalna G, . Improving the metabolic fidelity of cancer models with a physiological cell culture medium. Sci Adv 2019;5:eaau7314. 30613774 10.1126/sciadv.aau7314 PMC6314821

[47] Alderweireldt E , Grootaert C, De Wever O, Van Camp J. A two-front nutritional environment fuels colorectal cancer: perspectives for dietary intervention. Trends Endocrinol Metab 2022;33:105–19. 34887164 10.1016/j.tem.2021.11.002

[48] Dupuy F , Tabaries S, Andrzejewski S, Dong Z, Blagih J, Annis MG, . PDK1-dependent metabolic reprogramming dictates metastatic potential in breast cancer. Cell Metab 2015;22:577–89. 26365179 10.1016/j.cmet.2015.08.007

[49] Vasquez EG , Nasreddin N, Valbuena GN, Mulholland EJ, Belnoue-Davis HL, Eggington HR, . Dynamic and adaptive cancer stem cell population admixture in colorectal neoplasia. Cell Stem Cell 2022;29:1213–28. 35931031 10.1016/j.stem.2022.07.008 PMC9592560

[50] Cohen AS , Grudzinski J, Smith GT, Peterson TE, Whisenant JG, Hickman TL, . First-in-Human PET imaging and estimated radiation dosimetry of l-[5-^11^C]-glutamine in patients with metastatic colorectal cancer. J Nucl Med 2022;63:36–43. 33931465 10.2967/jnumed.120.261594 PMC8717201

[51] Amirkhah R , Gilroy K, Malla SB, Lannagan TR, Byrne RM, Fisher NC, . MmCMS: Mouse models’ consensus molecular subtypes of colorectal cancer. Biorxiv 2022:2022.06.17.496539. 10.1038/s41416-023-02157-6 PMC10050155 36717674

[52] Kratochwil C , Flechsig P, Lindner T, Abderrahim L, Altmann A, Mier W, . ^68^Ga-FAPI PET/CT: tracer uptake in 28 different kinds of cancer. J Nucl Med 2019;60:801–5. 30954939 10.2967/jnumed.119.227967 PMC6581228

[53] Flanagan DJ , Amirkhah R, Vincent DF, Gunduz N, Gentaz P, Cammareri P, . Epithelial TGFβ engages growth-factor signalling to circumvent apoptosis and drive intestinal tumourigenesis with aggressive features. Nat Commun 2022;13:7551. 36477656 10.1038/s41467-022-35134-3 PMC9729215

