# Supplementary material for: Noninvasive Stratification of Colon Cancer by Multiplex PET Imaging
Source: Clin Cancer Res. 2024 Mar 17;30(8):1518–29. doi: 10.1158/1078-0432.CCR-23-1063 (PMC11016897; doi:10.1158/1078-0432.CCR-23-1063)
Supplement: Supplementary Data 1 — Supplementary Figures S1-S8 [file ccr-23-1063_supplementary_data_1_suppfs1.pdf]

Graphical abstract

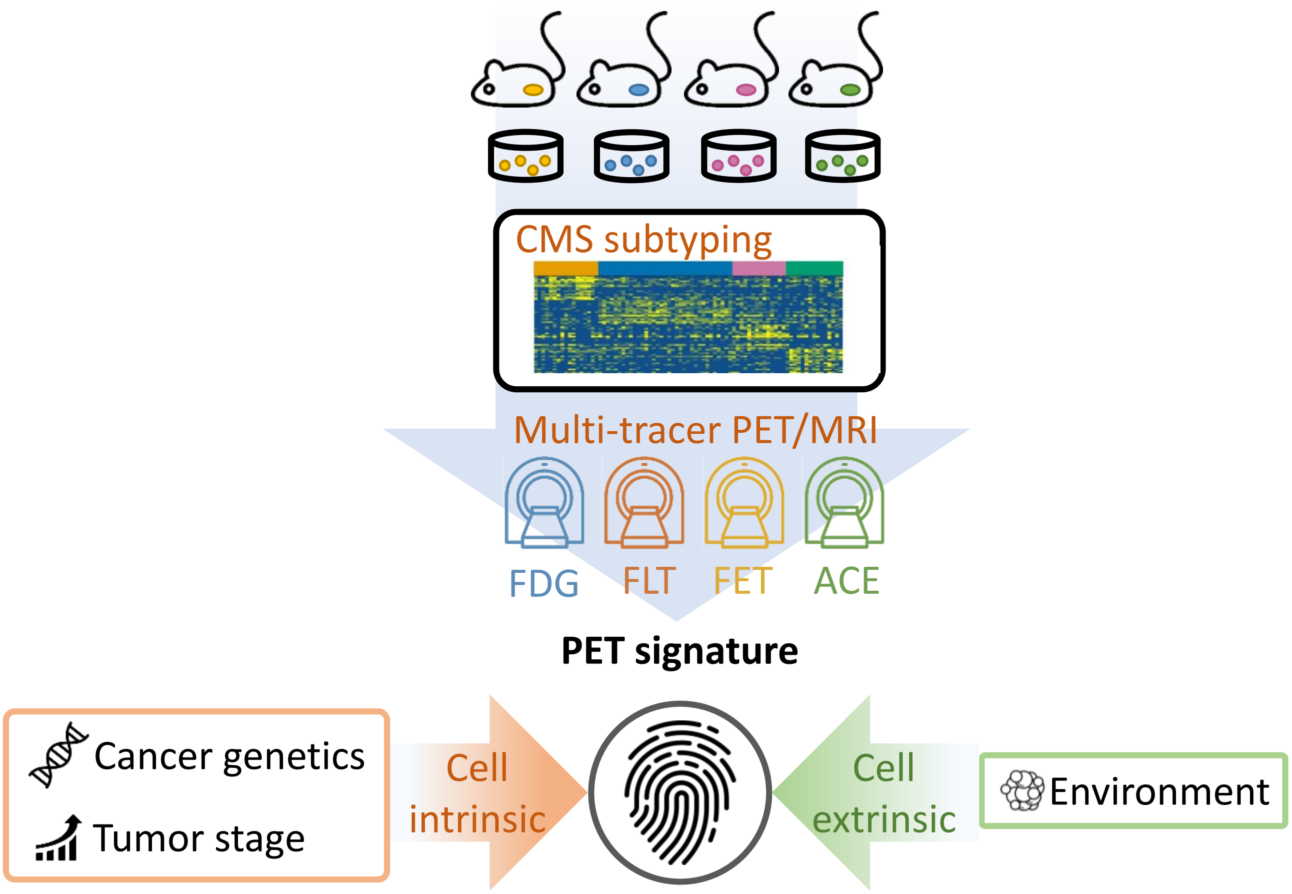

Figure S1

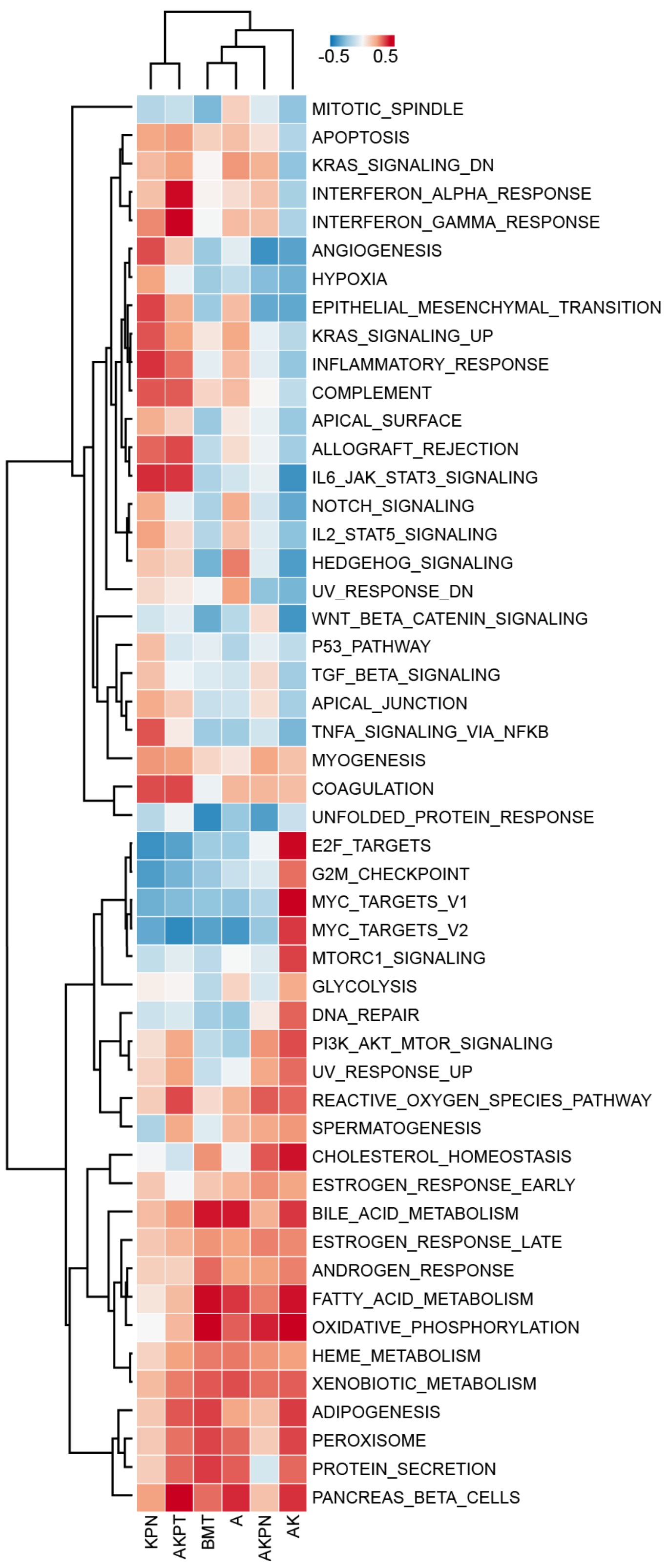

Figure S1 Extended heatmap illustrating correlation of intestinal cancer GEMMs' gene expression with the Molecular Signatures Database (MSigDB) hallmark gene set. An abridged version of this heatmap is shown in Figure 1e. A (n=5 mice), AK (n=1 mouse), AKPN (n=3 mice), AKPT (n=4 mice), KPN (n=18 mice), BMT (n=5 mice) for each genotype. Heatmap is grouped using hierarchical clustering, one minus Pearson correlation, average linkage on rows and columns.

Figure S2

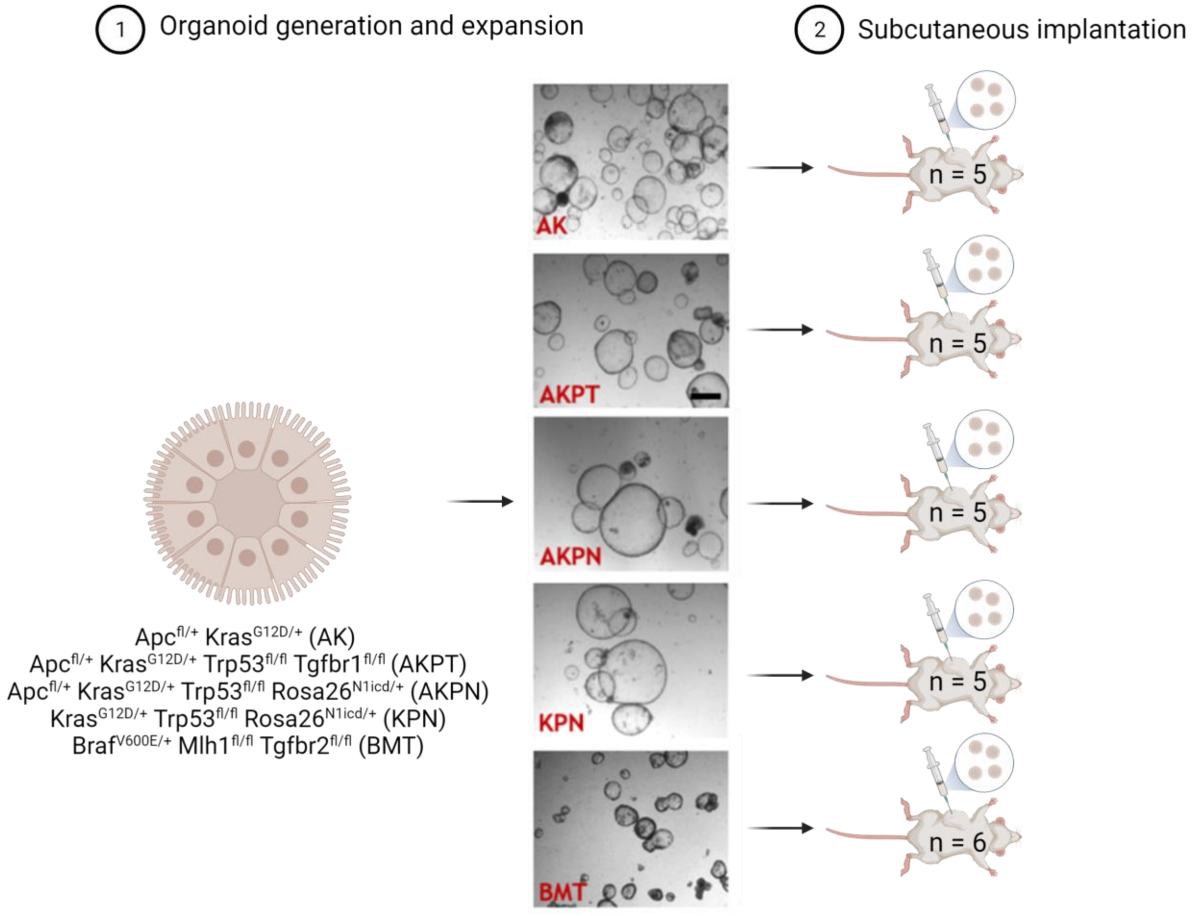

Figure S2 Schematic showing workflow for expanding and implanting organoids and white light microscopy images of resulting cultures. Black bar is 500 µm.

Figure S3

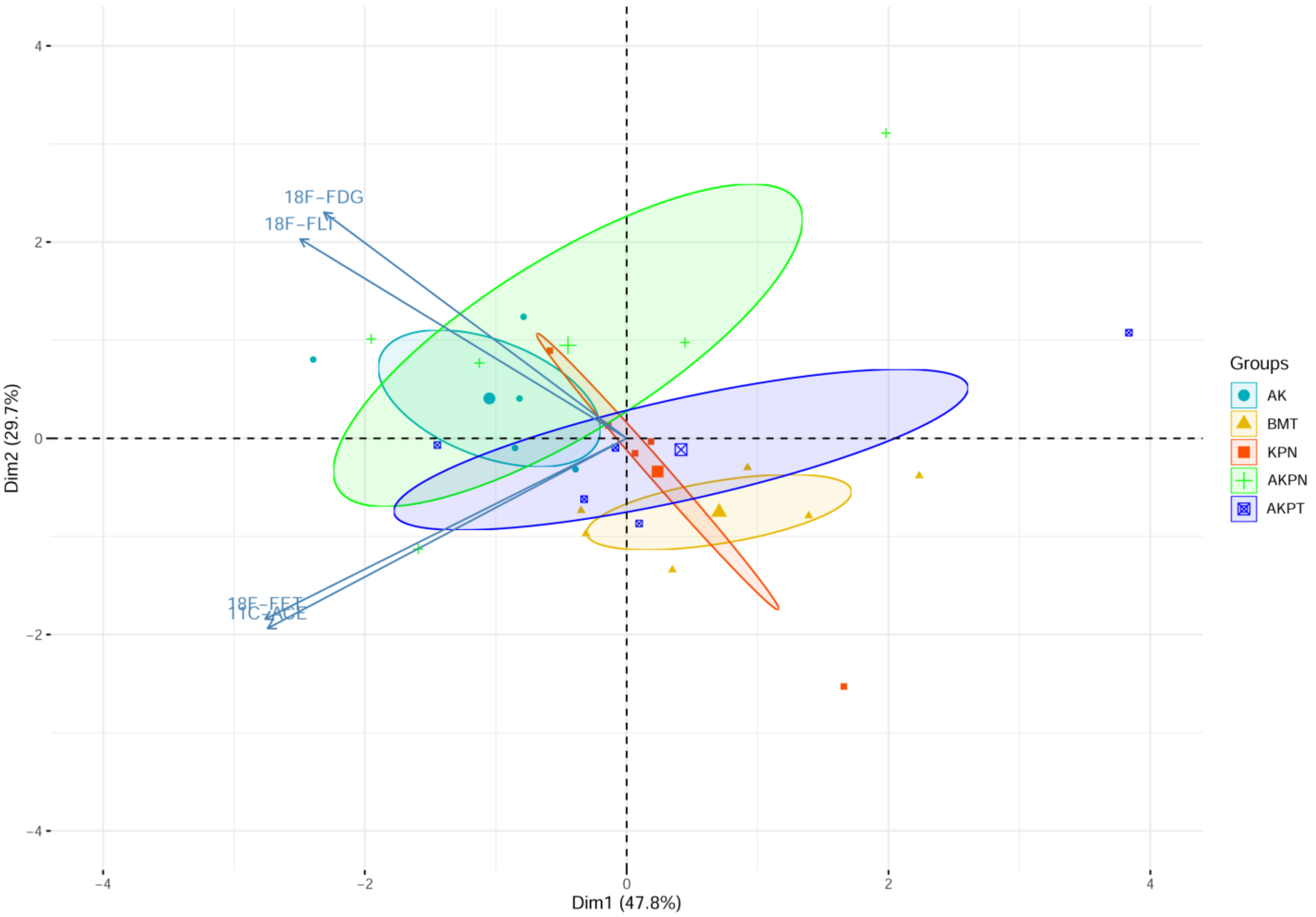

Figure S3 Principal component analysis biplot (PCA scores and loadings) from the 5 colon cancer mouse organoid subcutaneous models and 4 radiotracers used in this study. Groups are *Apcfl/+ KrasG12D/+* (AK), *BrafV600E/+ Mlh1fl/fl Tgfbr2fl/fl* (BMT), *KrasG12D/+ Trp53fl/fl Rosa26N1icd/+* (KPN), *Apcfl/+ KrasG12D/+ Trp53fl/flRosa26N1icd/+* (AKPN), and *Apcfl/+ KrasG12D/+ Trp53fl/fl Tgfbr1fl/fl* (AKPT). The radiotracers were [18F]Fluorodeoxy-D-glucose (FDG), O-(2-[18F]fluoroethyl)-L-tyrosine (FET), 3'-deoxy-3'- [18F]fluorothymidine (FLT), and [11C]acetate (ACE).

Figure S4

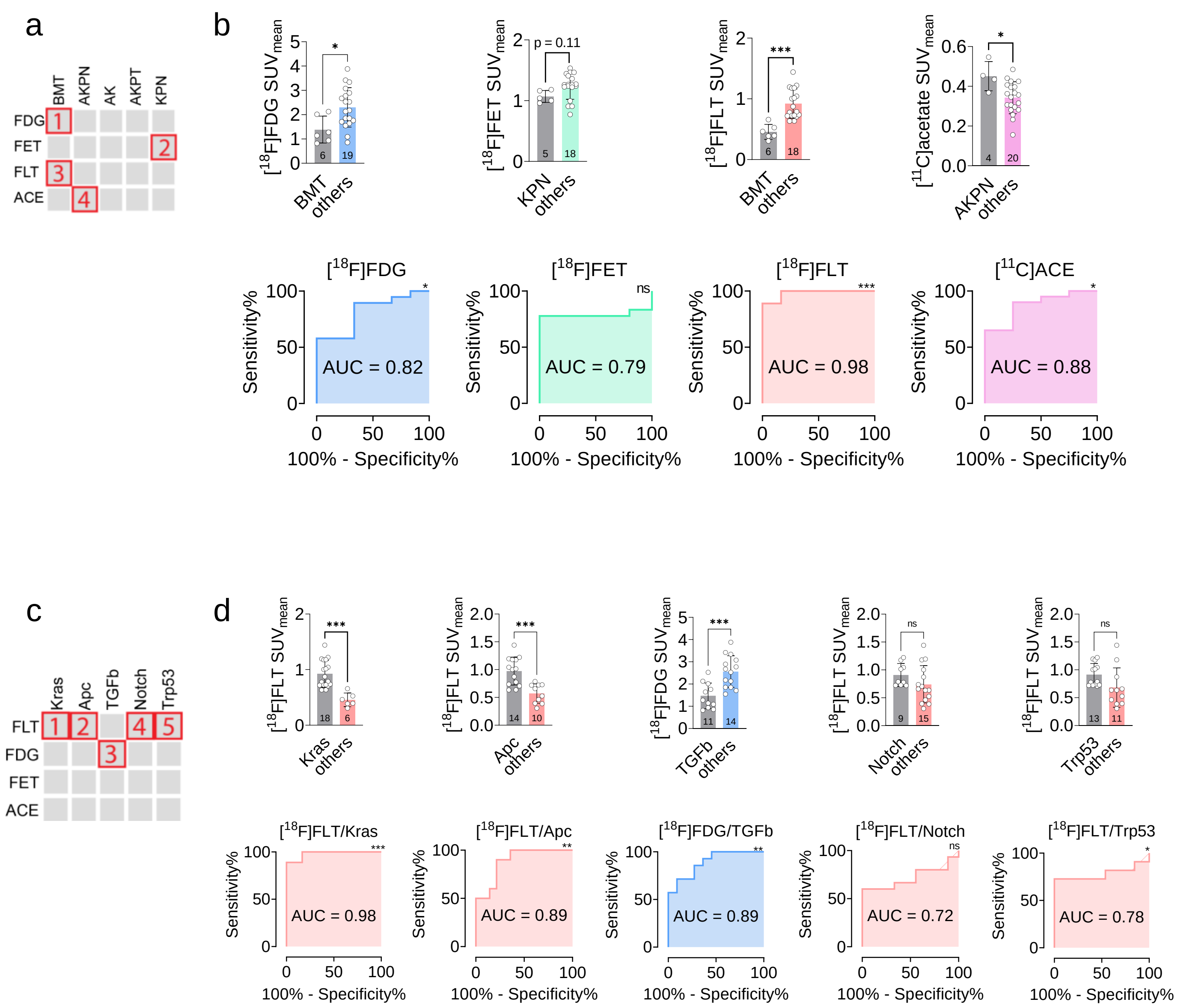

Figure S4. (a) Representation of separation matrix in Figure 3b, red boxes highlight PET radiotracers and models with the highest AUC (b) Boxplots and receptor operator characteristic (ROC) curves aligning to red boxes in a. Error bars represent standard deviation. The first set, [18F]FDG/BMT, is repeated here from Figure 3b for comparison. (c) Representation of separation matrix in Figure 3d, red boxes highlight PET radiotracers and genes with the clearest separation (d) Boxplots and ROC curves aligning to red boxes in 3c. Error bars represent standard deviation. The first set, [18F]FLT/Kras, is repeated here from Figure 3d for comparison. In total, 26 mice were implanted, each point in panels b and d represents a single mouse, some mice were not imaged with every tracer due to an earlier mouse death. * $p < 0.05$, ** $p < 0.01$, *** $p < 0.001$ for unpaired t tests and area under the ROC curves.

Figure S5

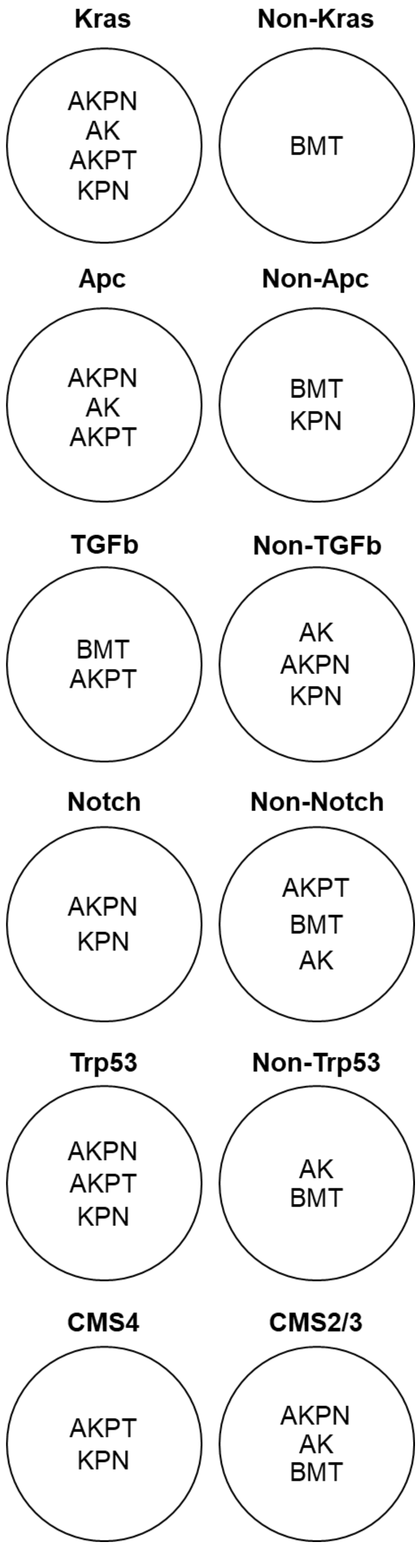

Figure S5. Grouping of the subcutaneous organoid colon cancer models according to genotype and consensus molecular subtype. Mice were pooled into different groups as shown to perform binary classification analysis using receptor operator characteristic curves. See also Figure 3 and S4.

Figure S6

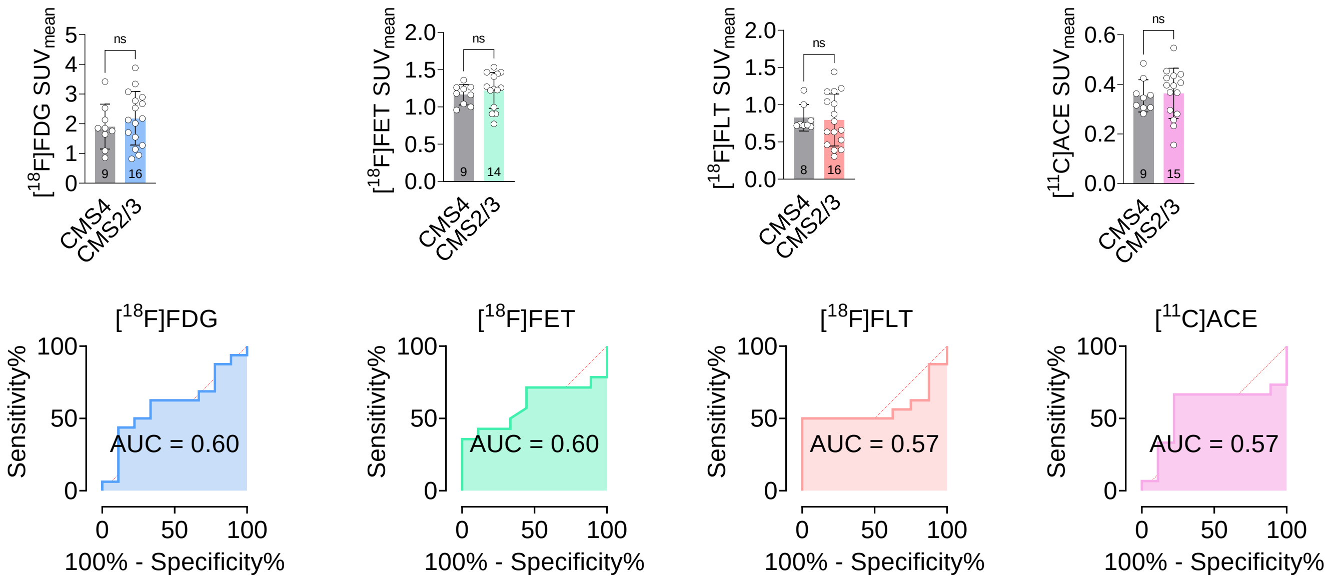

Figure S6. Boxplots and receiver operator characteristic curves for between consensus molecular subtype groups CMS4 and CMS2/3 with each radiotracer. Error bars represent standard deviation. Data compared by unpaired t test. In total, 26 mice were implanted, each point in top row of graphs represents a single mouse. Numbers inside bars show n. Some mice were not imaged with every tracer due to mouse death or tumour ulceration. ns, not statistically significant.

Figure S7

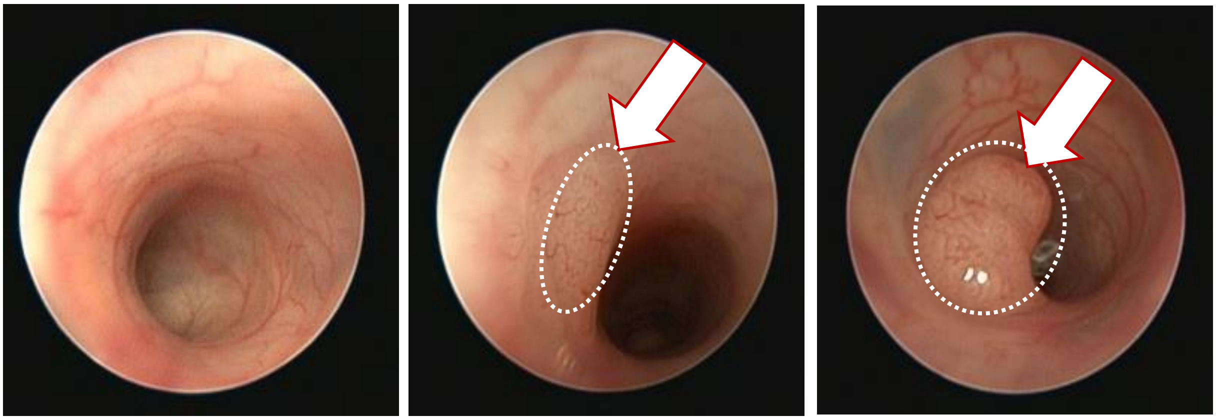

Normal colon | 2 week post-induction | 4 week post-induction

Figure S7 Tumour monitoring of orthopic colon tumors with colonoscopy. The arrow and white dotted line show the growing tumor. Colonoscopy not performed on GEMMs or subcutaneous organoids models.

Figure S8

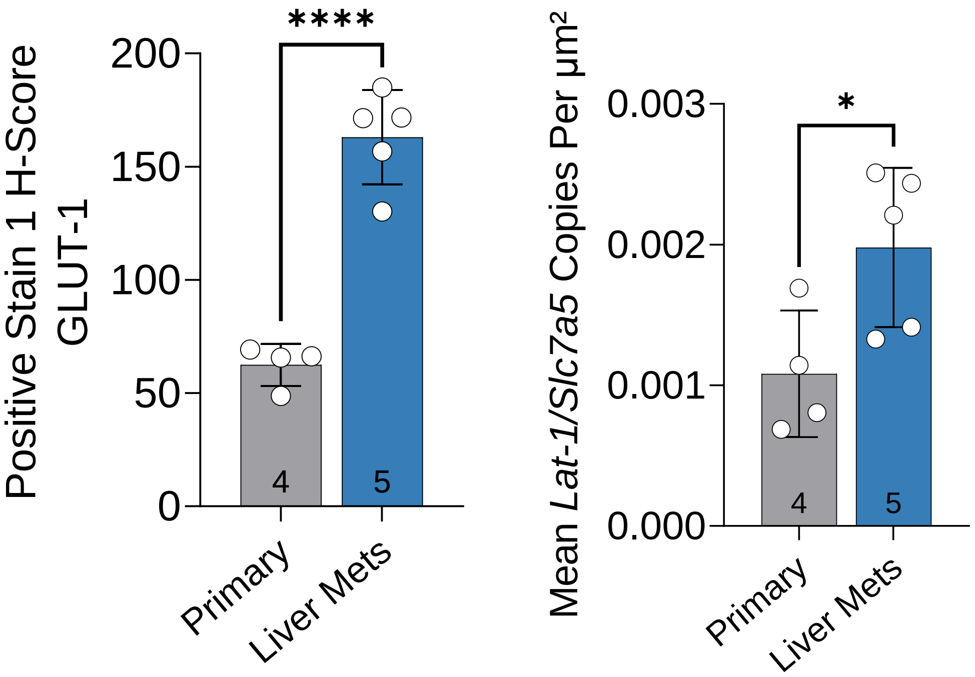

Figure S8. H-score of GLUT-1 immunohistochemistry and mean *Lat-1/Slc7a5* copies per µm^2 *in situ* hybridization of images in Figure 5d. Sample size (n) shown within bars. * $p < 0.05$, **** $p < 0.0001$. Data compared using unpaired t test.
